# A Transcript Profiling Approach Reveals an Abscisic Acid-Specific Glycosyltransferase (UGT73C14) Induced in Developing Fiber of *Ligon lintless-2* Mutant of Cotton (*Gossypium hirsutum* L.)

**DOI:** 10.1371/journal.pone.0075268

**Published:** 2013-09-23

**Authors:** Matthew K. Gilbert, John M. Bland, Jay M. Shockey, Heping Cao, Doug J. Hinchliffe, David D. Fang, Marina Naoumkina

**Affiliations:** 1 Cotton Fiber Bioscience Research Unit, USDA-ARS, Southern Regional Research Center, New Orleans, Louisiana, United States of America; 2 Food Processing and Sensory Quality Research Unit, USDA-ARS, Southern Regional Research Center, New Orleans, Louisiana, United States of America; 3 Commodity Utilization Research Unit, USDA-ARS, Southern Regional Research Center, New Orleans, Louisiana, United States of America; 4 Cotton Chemistry and Utilization Research Unit, USDA-ARS, Southern Regional Research Center, New Orleans, Louisiana, United States of America; New Mexico State University, United States of America

## Abstract

*Ligon lintless-2*, a monogenic dominant cotton (*Gossypium hirsutum* L.) fiber mutation, causing extreme reduction in lint fiber length with no pleiotropic effects on vegetative growth, represents an excellent model system to study fiber elongation. A UDP-glycosyltransferase that was highly expressed in developing fibers of the mutant *Ligon lintless-2* was isolated. The predicted amino acid sequence showed ~53% similarity with Arabidopsis UGT73C sub-family members and the UDP-glycosyltransferase was designated as UGT73C14. When expressed in *Escherichia coli* as a recombinant protein with a maltose binding protein tag, UGT73C14 displayed enzymatic activity toward ABA and utilized UDP-glucose and UDP-galactose as the sugar donors. The recombinant UGT73C14 converted natural occurring isoform (+)-cis, trans-ABA better than (+)-trans, trans-ABA and (-)-cis, trans-ABA. Transgenic Arabidopsis plants constitutively overexpressing UGT73C14 did not show phenotypic changes under standard growth conditions. However, the increased glycosylation of ABA resulted in phenotypic changes in post-germinative growth and seedling establishment, confirming *in vivo* activity of UGT73C14 for ABA. This suggests that the expression level of UGT73C14 is regulated by the observed elevated levels of ABA in developing fibers of the *Li*_*2*_ mutant line and may be involved in the regulation of ABA homeostasis.

## Introduction

Cotton is the major source of renewable fiber in the world, used primarily for a wide range of textile applications. Increased competition with international producers and synthetic fibers is forcing breeders to continually improve cotton fiber characteristics such as yield, length, strength and fineness. Genetic engineering has provided powerful tools for the improvement of cotton. However, the lack of information at the molecular level regarding genes and regulatory elements that control fiber development is one of the major limitations in the genetic improvement of cotton fiber.

Cotton fiber mutants are valuable tools for understanding the biological processes of fiber development. In cotton several fiber-related mutants have been discovered, one of which is the monogenic and dominant *Ligon lintless-2* (Li_*2*_), which exhibits an extreme reduction in the length of lint fiber [1]. *Li*_*2*_ mutant plants display normal vegetative growth, indicating no pleiotropic effects as a result of the mutation. Cytological studies have not revealed differences in seed fiber initiation between mutant and wild-type (WT) plants suggesting the effects of the mutation occurs later in development, likely during the elongation stage [2,3]. Therefore, the *Li*_*2*_ mutant in a near-isogenic state with a wild-type represents a good model system to study fiber elongation.

Plant hormones play important roles in fiber development and are considered to be targets for genetic manipulation [4]. It is well documented that exogenous applications of auxins and gibberellic acid stimulate the differentiation of fibers and promote elongation, while abscisic acid (ABA) and cytokinins inhibit fiber growth in an *in vitro* cotton ovule culture system [5,6]. Genetic manipulation of auxin biosynthesis in cotton ovule epidermal cells enhanced fiber yield and quality [7]. Lower levels of endogenous indole-3-acetic acid, zeatin, and gibberellic acid were determined in the fiberless (*fl*) mutant line, whereas increased level of ABA was detected during fiber initiation [8]. High levels of ABA and cytokinins were also detected in ovules and the developing fibers of a *Ligon lintless* (*Li*, it was not specified *Li*_*1*_ or *Li*_*2*_) mutant line [9].

The UDP glycosyltransferases (UGT) represent a superfamily of enzymes catalyzing transfer of the glycosyl group from a nucleotide sugar donor to a small hydrophobic molecule [10]. The nucleotide sugar donor is usually UDP-glucose, however UDP-galactose, UDP-glucuronic acid, UDP-xylose, and UDP-rhamnose are also used in plants. Acceptors comprise a vast diversity of molecular structures, including hormones and secondary metabolites which are synthesized in plant species. Glycosylation can result in the formation of poly-glycosides, disaccharides, and various mono-glycosides of various acceptor molecules. Plants contain a large number of the members of UGT the superfamily. In Arabidopsis 120 UGT-encoding sequences have been identified [11]. Phylogenetic comparisons of UGTs from plants, animals, fungi, bacteria, and viruses revealed that plant UGTs represent a distinct clade [11]. The plant UGTs are known to be involved in plant natural product synthesis, control of plant hormone homeostasis, and detoxification of xenobiotics [11-14]. These plant UGTs have an expanded UGT-defining sequence, denoted as the plant secondary product glycosyltransferase (PSPG) motif [15,16].

Glycosylation reactions serve to convert reactive aglycones into more stable and non-reactive storage forms. In addition, attachment of the hydrophilic glucose moiety to hydrophobic aglycones increases water solubility. Glycosylation by UGT is often the last step in the biosynthesis of natural products in plants [10,12,13]. The glycosylation reaction is also a key step in general detoxification mechanisms for xenobiotics in higher plants [10,17]. The biological function of the glycosylation step in plants is therefore to facilitate storage and intracellular transport. Glycosylation also serves as a regulatory step in homeostasis of plant growth regulators.

Despite the importance of hormone regulation in cotton fiber development processes, to date, no cotton UGT involved in these processes has been characterized at the molecular level. The aim of this study was to find and characterize *G. hirsutum* UGTs involved in the regulation of cotton fiber development. Using a transcript profiling and substrate screening approach, we identified UGT73C14 from cotton (*G. hirsutum*) that was able to glycosylate abscisic acid (ABA) *in vitro. In vivo* studies confirmed activity of this UGT against ABA, suggesting a role of UGT73C14 in ABA homeostasis in cotton.

## Results

### Selection of target UGT

**Figure 1:**
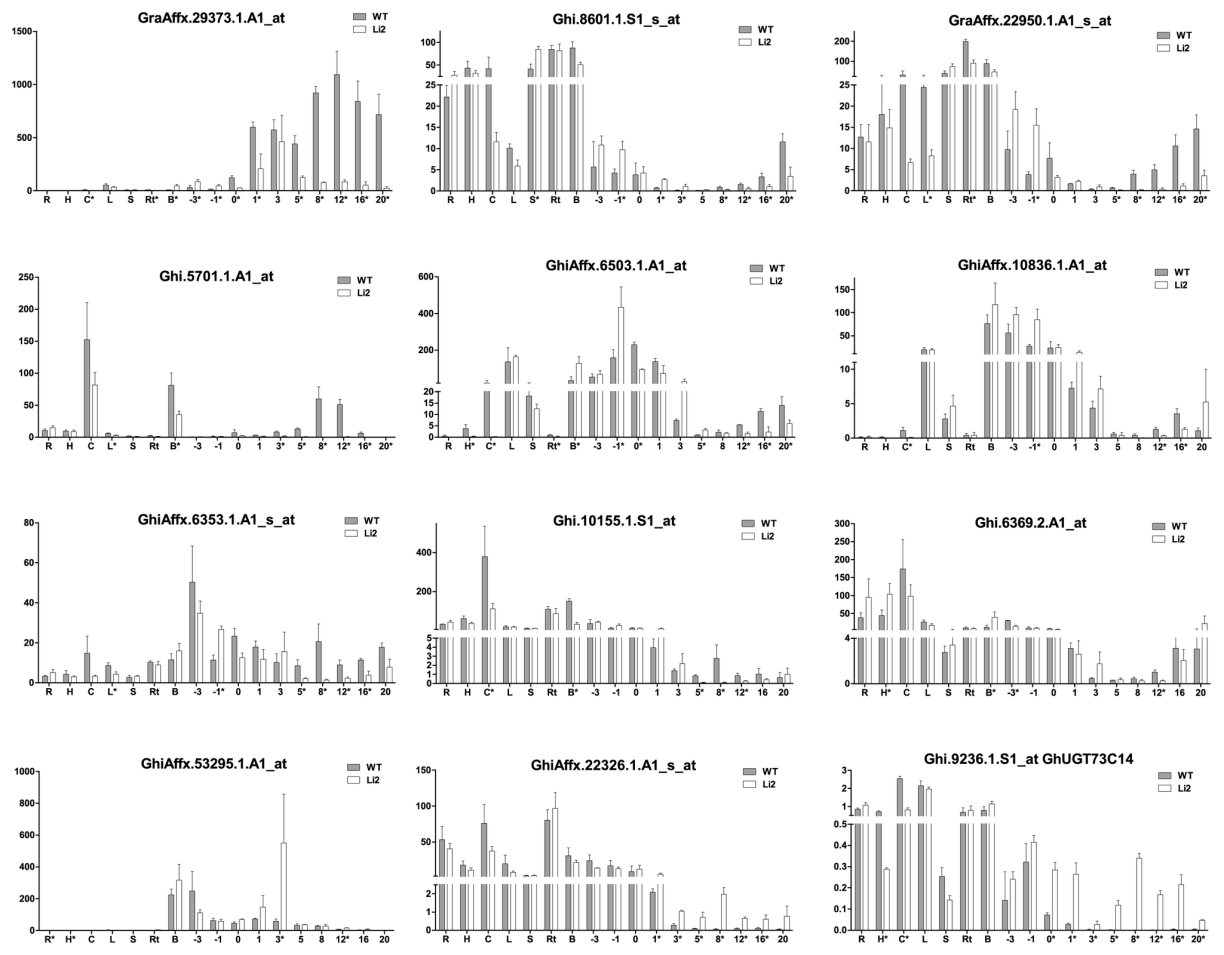
RT-qPCR analysis of expression level of putative UGTs from *Gossypium hirsutum*. Abbreviations: R, radicle; H, hypocotyls; C, cotyledon; L, leaf; S, stem; Rt, root; B, bud. Enriched epidermal cell fraction of ovules at -3, -1 and 0 DOA; fiber cells at 1, 3, 5, 8, 12, 16 and 20 DPA. Error bars indicate standard deviation from 3 biological replicates. Asterisks indicate significant different expression level between wild type and *Li*_*2*_ NILs.

A cotton Affymetrix microarray was used to perform gene expression profiling on fiber samples at three developmental time points, DOA, 8 DPA and 12 DPA, representing initiation and the peak of elongation fiber development stages [3]. Sequences of probe sets were screened for the presence of PSPG motif to determine UGT candidates represented on the microarray. Of the 21,854 probe sets consensus sequences, 43 contained the PSPG motif (Figure S1), and an additional 32 probe sets not identified in the motif scan were annotated as putative UDP-glycosyl transferases. Of these, microarray data indicated 13 probe sets were significantly differentially regulated in at least one evaluated time point (Table 1). RT-qPCR analysis was performed to evaluate expression levels of these putative UGTs in multiple cotton tissues, including root, leaf, stem, bud, radicle, hypocotyl, cotyledon, epidermal cells of ovule, and fiber at different developmental stages. RT-qPCR analysis confirmed that all selected UGTs were differentially regulated in fiber tissue of the *Li*_*2*_ mutant compared to WT (Figure 1).

**Table 1:** Affymetrix microarray data shows the expression ratios of selected putative UGTs.

**Probesets ID**	**DOA *Li2*/WT**	**8DPA *Li2*/WT**	**12DPA *Li2*/WT**
Ghi. 10155.1.S1_at	1.29	**0.47**	**5.94**
Ghi. 5701.1.A1_at	1.32	**0.11**	**0.05**
Ghi. 6369.2.A1_at	0.90	1.40	**13.98**
Ghi. 8601.1.S1_s_at	1.13	0.92	**4.21**
Ghi. 9236.1.S1_at	1.09	**13.35**	1.81
Ghi. 3235.1.A1_at	1.39	**38.61**	**4.28**
GhiAffx. 10836.1.A1_at	1.39	**2.29**	**8.18**
GhiAffx. 22326.1.A1_s_at	1.09	**2.12**	1.22
GhiAffx. 53295.1.A1_at	1.24	1.19	**3.07**
GhiAffx. 6353.1.A1_s_at	0.80	**0.40**	0.75
GhiAffx. 6503.1.A1_at	0.89	**4.11**	0.64
GraAffx. 22950.1.A1_s_at	1.14	0.90	**4.03**
GraAffx. 29373.1.A1_at	1.05	0.96	**0.35**

Microarray data at ≥ 2-fold difference (p-value < 0.05) in transcript abundance are shown in boldface and underlined.

A majority of the probe sets selected for RT-qPCR analysis showed unique expression profiles except Ghi.9236.1.S1_at/Ghi.3235.1.A1_at and GraAffx.22950.1.A1_s_at/Ghi.8601.1.S1_s_at, which showed very similar expression patterns with high correlation coefficients (r=0.96 and r=0.85 respectively; Figure S2). These results suggest they likely correspond to the same gene or to different genes involved in the same pathway or cellular function. We did not include transcript data of Ghi.3235.1.A1_at in Figure 1 due to its similar pattern with Ghi.9236.1.S1_at.

Probe set GraAffx.22950.1.A1_s_at was designed from an EST of *G* *. raimondii* , whereas Ghi.8601.1.S1_s_at came from *G. hirsutum*; however, the overlapping region of the probe sets showed 98% sequence identity. The putative UGTs represented by these probe sets showed the highest expression levels in root, bud, and stem of WT and mutant, whereas expression in WT and mutant fiber tissues were relatively low. The expression level in fiber gradually increased during fiber development reaching maximum levels at 20 DPA. In contrast to these UGTs, the putative UGT represented by probe set GraAffx.29373.1.A1_at was expressed primarily in fiber tissue, although very low expression level was detected in leaf tissue. Compared to WT fibers, the *Li*_*2*_ mutant fibers showed significantly lower transcript abundance during the elongation and transition stages. The expression pattern of this putative UGT, preferentially in fiber tissue, suggests it also could have role in cotton fiber development.

Another interesting expression pattern was observed for the Ghi.5701.1.A1_at probe set. This UGT was highly expressed in cotyledon and bud tissues of WT and mutant, whereas its expression level gradually increased in elongating fibers of WT, reaching a maximum at 8 DPA followed by reduced transcript abundance at the SCW biosynthesis stage. The transcript abundance of this UGT was significantly lower in fibers of the *Li*_*2*_ mutant compared to WT fibers during the elongation stage. This expression pattern suggests involvement of UGT (Ghi.5701.1.A1_at) in the elongation process of cotton fiber. The putative UGT represented by probe set GhiAffx.6503.1.A1_at showed a high expression level in epidermal cells of ovules during fiber initiation (-3 DOA -1 DPA) and leaf tissue in WT plants, whereas this UGT was up-regulated 3-4 times in *Li*_*2*_ mutants at -1DOA and bud tissue. Low expression levels for this putative UGT were detected in fiber tissue; however, it gradually increased from elongation to SCW deposition in WT with significant reduction of expression levels in *Li*_*2*_ mutant fibers. The putative UGT corresponding to GhiAffx.10836.1.A1_at was highly expressed in bud and leaf tissues and epidermal cells of ovules (-3 to 0 DOA) of WT and mutant, while its expression was significantly lower during the elongation stage of fiber development in *Li*_*2*_ fibers compared to WT fibers.

The putative UGT (GhiAffx.6353.1.A1_s_at) showed the highest expression level in epidermal cells of ovules and developing fibers and was also expressed in cotyledon, leaf, bud, and root. In *Li*_*2*_ mutant, the transcript level of this UGT was significantly up-regulated during initiation at -1 DOA, but down-regulated during elongation at 5-16 DPA. Ghi.10155.1.S1_at and Ghi.6369.2.A1_at showed the highest expression in cotyledon, while low transcript levels were detected in epidermal cells of ovules and even lower levels in developing fibers. In the *Li*_*2*_ line, Ghi.10155.1.S1_at was significantly down-regulated in cotyledon, bud, and elongating fiber at 5-12 DPA, whereas Ghi.6369.2.A1_at was up-regulated in hypocotyl and bud and showed reduced transcript level in -3 DOA and 12 DPA fibers compared to WT fibers. The highest transcript level for GhiAffx.53295.1.A1_at was present in bud, epidermal cells of ovules, and fiber tissue, and was significantly up-regulated in *Li*_*2*_ developing fiber at 3 DPA compared to WT fibers.

UGTs (GhiAffx.22326.1.A1_s_at and Ghi.9236.1.S1_at) showed the most interesting expression profiles. In WT plants they expressed in all tested tissues except the developing fiber, whereas these putative UGTs were induced in fiber cells only in the *Li*_*2*_ line. Induction of UGTs in major organs and during different developing stages can be related to the glycosylation of metabolic compounds such as hormones involved in regulation of plant developmental processes. Characterization of a UGT induced only in the short fibers of the *Li*_*2*_ mutant line will provide insight on the physiology of fiber elongation and the role of hormonal regulation. For this reason we selected the putative UGTs (Ghi.9236.1.S1_at/Ghi.3235.1.A1_at), which were both up-regulated approximately 100-200 times in *Li*_*2*_ elongating fibers compared to WT fibers (Figure 1), for further functional analysis.

### UGT isolation, sequence and copy number analysis

Sequences from the probe sets consensus sequences Ghi.9236.1.S1_at and Ghi.3235.1.A1_ at were used for primer design to conduct RACE-PCR. Recovered full length sequences revealed 100% identity, confirming that the probe sets correspond to the same gene. The open reading frame of this UGT contains 1,449 bp, which translates into a 482 amino acid peptide. PCR and agarose gel electrophoresis using cDNA and genomic DNA determined there are no introns in the gene (data not shown). Subjecting the sequence to non-redundant BLASTP analysis revealed relatively low similarity to previously characterized plant UGTs. The highest homology (around 53%) showed Arabidopsis UGT73C sub-family members (Figure S3). The UGT was designated as UGT73C14 by Nomenclature Committee [15].

**Figure 2:**
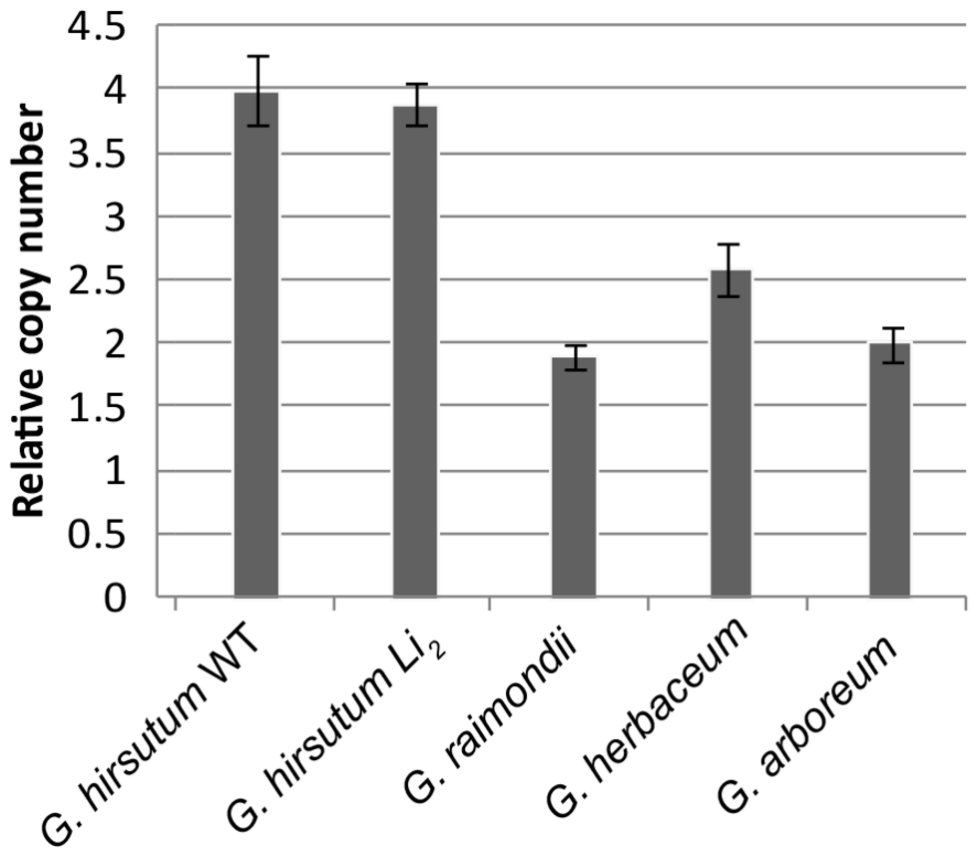
Copy Number Variation Assay of UGT73C14 using the single copy GhMYB25 as a reference gene. Genomic DNA was isolated from frozen leaf tissues using Qiagen DNeasy DNA extraction kit, and subjected to a 5 cycle Specific Template Amplification (STA) reaction. The STA reaction product was then subject to Taqman digital PCR following the manufacturer’s recommendations and microfluidic chips provided by Fluidigm. A ratio of UGT73C14 positive wells to GhMYB25 positive wells determined the relative copy number. To determine the copy number (y-axis values), for tetraploid lines (G. *hirsutum*), the UGT73C14/GhMYB25 ratio was multiplied by four, and for diploid lines (G. *raimondii, G*. *herbaceum* and *G*. *arboreum*), the UGT73C14/GhMYB25 ratio was muliplied by two. Error Bars represents the 95% Confidence Interval.

To further characterize UGT73C14 we utilized a Fluidigm microfluidic dPCR assay to determine gene copy number in G. hirsutum (AD-genome), G. *ramondii* (D_5_ genome), G. *herbaceum* (A_1_ genome), and G. *arboreum* (A_2_ genome) (Figure 2). GhMYB25 was used as a control as it has been previously demonstrated to be present as a single-copy gene in diploid species and a two-copy gene in the tetraploid genome [18]. The results indicate that UGT73C14 is present as a single copy per haploid genome – a total of 4 copies in the tetraploid *hirsutum* and 2 copies each in the diploid species *G. arboreum* *,* *G* *. herbaceum* , and *G* *. raimondii* . Furthermore, there was no difference in copy number between the *Li*_*2*_ mutant line and WT.

### Phylogenetic Analysis

**Figure 3:**
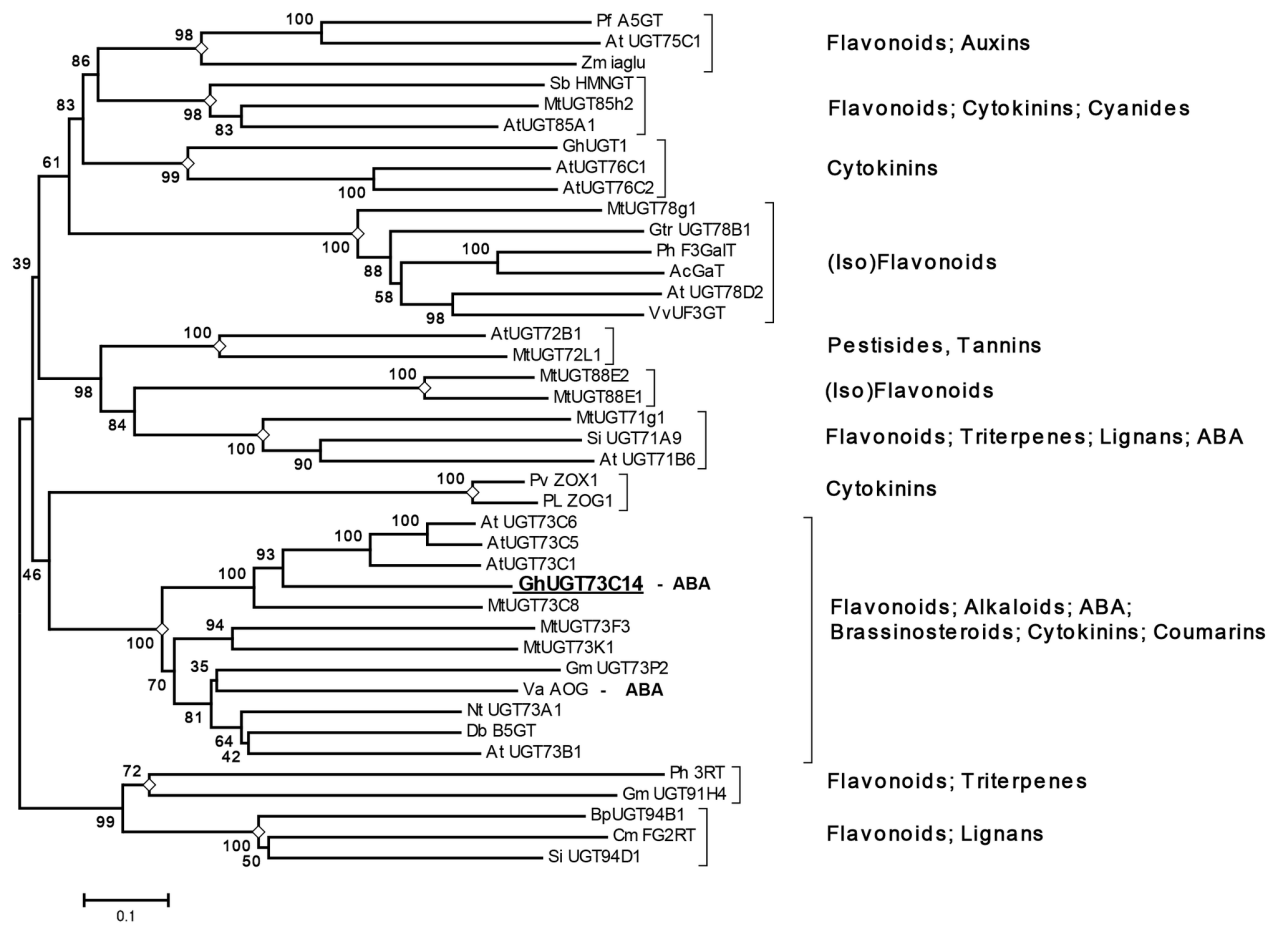
Phylogenetic analysis of functionally characterized plant glycosyltransferases and sequence comparison of zeatin specific binding site. Phylogenetic analysis was conducted in MEGA5 [56] using the Neighbor-Joining method [57]. Accession numbers, references and details regarding substrate acceptors, donors and products for the UGT’s used in phylogenetic analysis are provided in Table S1. Taxon names include abbreviation of species and UGT systematic name (or alternative name if nomenclature name is not available). The amino acid sequences were aligned using MUSCLE program [58]. The bootstrap consensus tree inferred from 1000 replicates [59] represents the evolutionary relationship of the UGTs. Branches corresponding to partitions reproduced in less than 50% bootstrap replicates are collapsed. The percentage of replicate trees in which the associated UGT clustered together in the bootstrap test (1000 replicates) is shown next to the branches. The tree is drawn to scale with branch lengths in the same units as those of the evolutionary distances used to infer the phylogenetic tree. The evolutionary distances were computed using the Poisson correction method [60] and are in the units of the number of amino acid substitutions per site. The analysis involved 41 amino acid sequences. All positions containing gaps and missing data were eliminated. There were a total of 337 positions in the final dataset. Nodes representing different UGT families are labeled by empty diamonds. Classes of substrate acceptors are shown from right.

To explore the evolutionary relationship among biochemically characterized plant UGTs (Table S1) the Neighbor-Joining tree was constructed using full length amino acid sequences. A total of 337 amino acid positions were aligned for 41 UGTs to construct a phylogenetic tree (Figure S3). The analyzed UGT sequences were distributed into 11 groups (marked by empty diamonds) with each group containing the same class of UGT’s from different species, suggesting divergence of ancestral UGT’s before speciation occurred (Figure 3). Biochemical data has demonstrated that different members from the same class of UGTs have evolved the ability to glycosylate a large number of structurally different aglycones *in vitro*. For example, members of class 73 that includes UGT73C14 show specificity to flavonoids, alkaloids, ABA, brassinosteroids, cytokinins, and coumarins [19-26]. Likewise, members from different classes showed specificity to these same substrate acceptors. For example, both Arabidopsis UGT85A1 and UGT73C1 are able to glycosylate different isoforms of zeatin [19]. The sequences with highest similarity to cotton UGT73C14 were Arabidopsis zeatin O-glucosyltransferases UGT73C1 and UGT73C5, whereas ABA-specific AOG from adzuki bean shared only 33% identity.

### *In vitro* activity of UGT73C14

**Figure 4:**
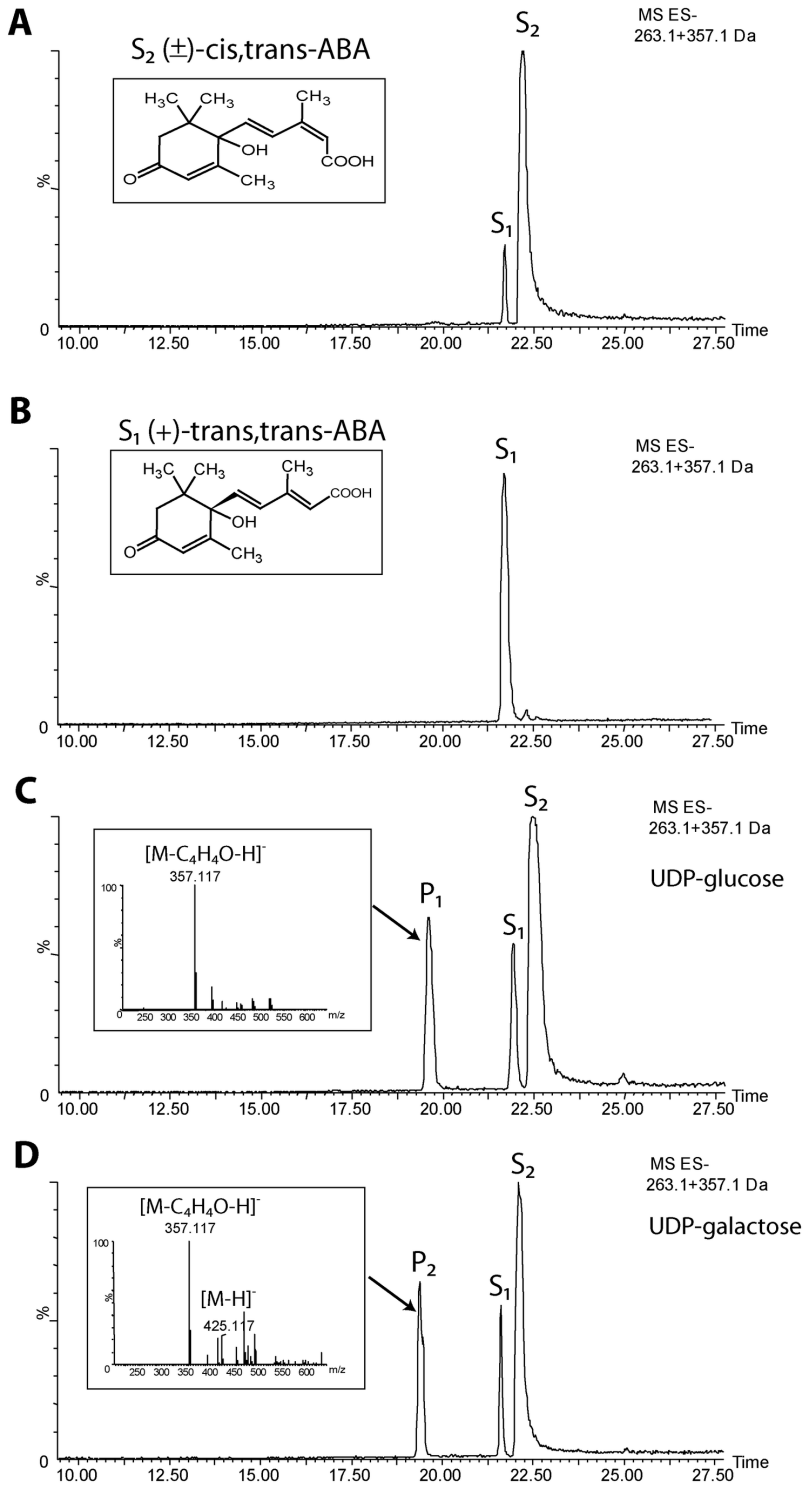
LCMS analysis of *in vitro* activity of UGT73C14 against ABA. Portions of negative LCMS extracted-ion chromatograms (m/z 263.1 + 375.1) show reaction products in the absence of enzyme (A) or with purified protein of UGT73C14 transferring UDP-glucose (C) or UDP-galactose (D) sugar-donors to the substrate acceptor - S. Panel B shows chromatogram of (+)-trans ,trans-ABA standard. S_1_ peak represents trans,trans-ABA isoform and S_2_ peak corresponds to mixture of (+)-cis,trans-ABA, whereas peaks of products P_1_ and P_2_ correspond to ABA-glucoside and ABA-galactoside, respectively. Insets represent MS spectra of ABA-glucoside (C) and ABA-galactoside (D).

The *G. hirsutum* UGT73C14 was expressed as a recombinant MBP-fusion protein in *E. coli* and purified by affinity chromatography (Figure S4). The purified protein was screened for *in vitro* activity against different metabolic compounds including auxins, cytokinins, gibberellic acid, abscisic acid, brassinolide, flavonols, hydroxycinnamates, coumarin, and salicylic acid (Figure S5) utilizing three commercially available nucleotide sugar donors: UDP-glucose, UDP-galactose, and UDP-glucuronic acid. UDP-sugars are the predominant nucleotide sugars in developing cotton fibers; UDP-glucose represents over 75% of the UDP-sugar fraction, while UDP-galactose and other UDP-sugars are also detectable [27,28]. We suggested potential involvement of UGT73C14 in hormone regulation since the closest Arabidopsis homologs UGT73C1 and UGT73C5 were active against of zeatin and brassinolide (Figure S3 and Table S1). Therefore a wide range of hormones (including those naturally present in plant and synthetic versions) were selected for screening activity of UGT73C14. Different classes of other compounds were included to estimate *in vitro* specificity of UGT73C14. Of these, only ABA was glycosylated by recombinant UGT73C14 utilizing UDP-glucose and UDP-galactose as the sugar donors. None of the other tested substrates were glycosylated by UGT73C14. The LCMS chromatograms of the reaction products are shown in Figure 4. Negative LCMS extracted-ion chromatogram (no enzyme was added to reaction, Figure 4A) shows two peaks in the commercially available ABA substrate, where S_1_ corresponds to trans, trans-ABA isoforms (matching the standard, Figure 4B) and S_2_ to (+)-cis,trans-ABA isoforms. Product was detected in enzymatic reactions utilizing UDP-glucose (Figure 4C, P_1_) or UDP-galactose (Figure 4D, P_2_) as sugar donor.

Kinetic analysis of UGT73C14 was performed with different isoforms of ABA. As shown in Table 2 UGT73C14 displayed typical Michaelis-Menten kinetics for (+)-cis, trans-ABA (UDP-glucose and UDP-galactose as sugar donors) and (+)-trans, trans-ABA (UDP-glucose as sugar donor). It displayed a sigmoidal rate-substrate concentration relationship with (+)-trans, trans-ABA utilizing UDP-galactose as sugar donor and (-)-cis, trans-ABA (UDP-glucose as sugar donor), whereas no activity was detected with (-)-cis, trans-ABA utilizing UDP-galactose as sugar donor. The enzyme exhibited the lowest K_m_ value (61.7 µM) and the highest substrate preference (K_cat_/K_m_ 115.1 s^-1^mM^-1^) for (+)-cis, trans-ABA utilizing UDP-glucose as sugar donor. Thus, *G. hirsutum* glycosyltransferase UGT73C14 showed *in vitro* preference toward the naturally present isoform of abscisic acid, (+)-cis, trans-ABA.

**Table 2:** Kinetic parameters for UGT73C14 toward isoforms of ABA.

**Sugar donor**	**Substrate**	**V_max_ (µmol/min**)	**K_m_ (µM**)	**K_cat_ (s^-1^**)	**K_cat_/K_m_ (s^-1^ mM^-1^**)
UDP-glu	(+)-cis, trans-ABA	221.3	61.7	7.2	115.1
UDP-gal	(+)-cis, trans-ABA	58.2	71.1	1.9	26.2
UDP-glu	(+)-trans, trans-ABA	82.9	98.1	2.7	27.1
UDP-gal	(+)-trans, trans-ABA	Displays a sigmoidal rate-substrate concentration relationship			
UDP-glu	(-)-cis, trans-ABA	Displays a sigmoidal rate-substrate concentration relationship			
UDP-gal	(-)-cis, trans-ABA	Not detected			

### *In vivo* activity of UGT73C14

**Figure 5:**
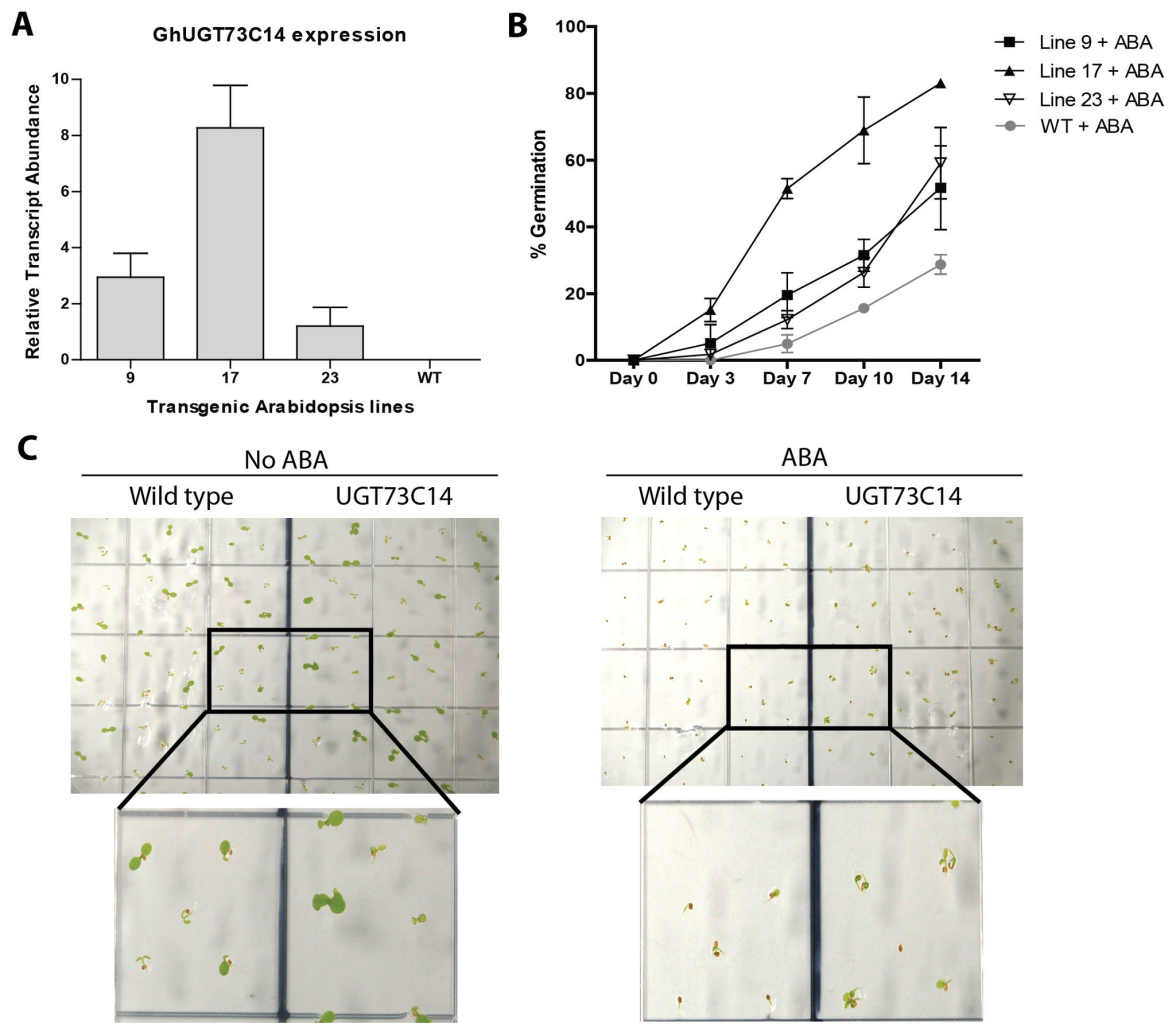
*In vivo* activity of UGT73C14 against ABA. (A) Relative transcript abundance of *G*. *hirsutum* UGT73C14 in transgenic Arabidopsis lines evaluated by RT-qPCR analysis. Error bars represent the standard deviation from three plants of line. (B) Transgenic *Arabidopsis* *thaliana* seeds overexpressing UGT73C14 (lines 9, 17, and 23) and wild type (Wt) were plated on phytagel supplemented with 2.2g/L Murashige and Skoog basal salts at pH 6.0. Each transgenic or wild type line was plated with and without filter sterilized 0.5μM Abscisic Acid in the phytagel. After 4 days at 4°C in darkness, germination occurred over 14 days in 8: 16 hr dark: light cycles at 18°C and 22°C, respectively. The percentage of germinated seedlings showing 2 emerged cotyledons was determined on days 0, 3, 7, 10 and 14. Statistical analysis of cotyledon emergence rate between transgenic and wild type plants is provided in Data S1. Error bars represent the standard deviation. (C) Representative picture of cotyledon emergence of wild type and UGT73C14 in the absence (left) or presence of 0.5 µM Abscisic Acid (right). The pictures were taken on day 6 after planting of T2 transgenic seeds of line 9 and wild type.

Arabidopsis transgenic lines were generated that constitutively over-expressed UGT73C14 under control of the CaMV 35S promoter. PCR screening with vector specific primers confirmed the presence of UGT73C14 in 27 transgenic lines (data not shown). The glycosylated forms of ABA are considered to be biologically inactive [29]. Constitutive glycosylation of ABA *in planta* by overexpression of the UGT could lead to phenotypic changes typical for ABA deficiency. However, we did not observe any visible phenotypic changes in transgenic plants compared to wild type under standard growth conditions. Exogenous application of ABA typically inhibits Arabidopsis seeds germination [30]. Therefore, over-expressing ABA-specific UGT may result in deactivation of the ABA inhibition effect and cotyledons of the transgenic lines would emerge more rapidly. Transgenic Arabidopsis seedlings were plated on phytagel containing 0.5μM ABA. Cotyledon emergence of both wild type and UGT73C14 transgenic lines was 100% after 6 days in the absence of ABA; however, in the presence of ABA the cotyledons of the transgenic lines emerged more rapidly. Figure 5 shows results of RT-qPCR analysis, the post-germinative growth curves and representative pictures of cotyledon emergence of Arabidopsis wild type and a transgenic line expressing UGT73C14. Of the seven lines tested, three showed a statistically significant higher percentage of seedlings germinating than non-transformed plants (Data S1). Three other lines also demonstrated higher germination rates than non-transformed plants, however the deviation was determined insignificant (data not shown).

## Discussion

Hormones play important roles in plant developmental processes and adaptation to environmental changes. Precise control of the level of different hormones in plant tissues is critical for plant survival and a wide range of regulatory mechanisms have evolved. Glycosylation is thought to be one of these mechanisms, and the majority of hormones exist as glycosides *in planta* [10,13]. To understand the role of glycosylation in cotton fiber developmental processes we analyzed microarray data in search of glycosyltransferases that are differentially regulated in the short fiber of *Li*_*2*_ mutants relative to its WT NIL. The non-biased expression and screening approaches were applied previously for identification of flavonoids and/or isoflavonoids glycosyltransferases [21]. Unlike classical methods, such as activity-based purification or sequence-based identification, this approach addresses the *in vivo* function which is difficult to determine *in vitro* due to redundant and promiscuous activity among the many members of the plant UGT superfamily. In the present study using a transcript profiling and substrate screening approach we identified UGT73C14 from cotton (*G. hirsutum*) that was able to glycosylate ABA *in vitro*. *In vivo* activity of this glycosyltransferase against ABA was confirmed by post-germinative growth of transgenic Arabidopsis plants over-expressing UGT73C14 on ABA-containing medium.

The enzyme UGT73C14 characterized in this study utilizes UDP-glucose and UDP-galactose as sugar donors for ABA. It was previously shown that the preference of plant UGTs for UDP-glucose or UDP-galactose is dependent on the nature of the last residue in the PSPG box, and changing this residue from histidine to glutamine in the *Aralia* *cordata* UDP-galactose: anthocyanin galactosyltransferase enabled the enzyme to utilize UDP-glucose as a sugar donor in addition to maintaining inherent galactosyltransferase activity [31]. As shown in our sequence alignment, UGT73C14 contains the glutamine residue at the last position in the PSPG box, whereas all other UDP-galactose specific UGTs possess histidine (Figure S3). The dual specificity determined by the *in vitro* assay suggests two possibilities: 1) the UDP-galactose is not a natural sugar donor for UDP73C14 or 2) the donor preference is more complex than suggested by the previous report [31]. It is possible that the galactosylation of ABA *in vitro* is an artifact since no galactosides of ABA have been reported in plant tissues.

ABA is a 15-carbon sequiterpenoid plant hormone and the naturally occurring form is (+)-cis, trans-ABA [32]. The ABA glucosyl ester is the most widespread conjugate which occurs through *O*-glucosylation at the C-1 hydroxyl group [33-36]. To date, enzymes mediating ABA glycosylation have been identified in different plant species. The first gene encoding an ABA UGT (AOG) was identified in adzuki bean, which was able to conjugate ABA with UDP-glucose [36]. The recombinant AOG converted (+)-trans, trans-ABA better than (+)-cis, trans-ABA and (-)-cis, trans-ABA. Also this enzyme showed weak activity with trans-cinnamic acid. On the other hand, AOG does not catalyze the conjugation of phaseic acid in bean plants. In Arabidopsis, eight recombinant UGTs were shown to glucosylate ABA *in vitro* and only one of them, UGT71B6, displayed a preference for the naturally occurring (+)-cis, trans-ABA [37]. In another study, analyses of the activity of UGT71B6 towards ABA and its structural analogues further confirmed that the enzyme preferentially glucosylated ABA, and not its catabolites [38]. K_cat_/K_m_ values are more informative for substrate specificity comparisons. The UGT73C14 showed activity toward three tested isoforms of ABA, including (+)-cis, trans-ABA, (+)-trans, trans-ABA and (-)-cis, trans-ABA. However, K_cat_/K_m_ value was four times higher toward the naturally occurring (+)-cis, trans-ABA isoform compared to (+)-trans, trans-ABA, whereas (-)-cis, trans-ABA displayed a sigmoidal rate-substrate concentration relationship (Table 2). Therefore, UGT73C14, unlike AOG from the adzuki bean, has higher *in vitro* activity to the naturally occurring ABA isoform. Also, in contrast to previously characterized enzymes from the adzuki bean and Arabidopsis, UGT73C14 utilized two sugar donors for ABA conjugation.

No phenotypic changes under normal growth conditions were reported for overexpression of ABA-specific Arabidopsis UGT71B6 [39]. However, in the presence of ABA on germination medium the cotyledons of transgenic UGT73C14 plants emerged more rapidly, suggesting a ABA deactivation role and therefore reduced impact of ABA on seedling establishment. Our results showed similar post-germinative growth effect of Arabidopsis transgenic lines over-expressing UGT73C14, suggesting *in vivo* the role of this UGT in the glycosylation of ABA.

ABA plays important roles in many biological processes including seed development, dormancy, germination, vegetative growth, and environmental stress responses [32]. ABA-glucosides exhibit little or no biological activity, but appear to be a transported form of ABA [40]. In general, leaves have a higher ABA content than roots, and developing seeds and ripening fruits are usually rich sources of ABA [41]. Consistent with the accumulation of ABA in plant organs, the expression level of UGT73C14 was high in cotyledon and leaf tissues of wild type plants (Figure 1). The inhibitory effect of ABA on cotton fiber development has also been well-documented, as the increased level of ABA inhibits cotton fiber cell elongation in the *in vitro* cotton ovule culture system [5,6]. In plants, extremely high ABA levels were measured on the first day post anthesis in a *Li* mutant (authors did not specify if *Li*_*1*_ or Li_2_ was examined) compared to wild type [9]. The results confirming *in vivo* ABA-activity coupled with evidence of high accumulation of ABA in mutant fiber suggests a possible role of UGT73C14 for ABA homeostasis in the *Li*_*2*_ mutant fibers. Elevated expression levels of UGT73C14 in the fibers of the mutant *Li*_*2*_ line could likely be a feedback response to altered hormone levels, acting to “detoxify” fiber cells by targeting excess hormones for storage.

Plant hormones are considered for genetic manipulations to improve cotton fiber quality. Down regulation of ABA may contribute to a longer period of fiber elongation. Comparative genetical genomics analysis of *G. hirsutum* and *G* *. barbadense* revealed that the down-regulated expression of ABA signaling pathway genes at the fiber developmental transition stage in *G* *. barbadense* may account for superior fiber qualities [42]. The glycosylation is often the last step in many biosynthetic pathways, thus manipulation at this step reduces the potential for producing unintended deleterious effects. Over-expressing ABA-specific UGT under a tissue specific promoter may reduce ABA activity and provide the same effect on fiber elongation. UGT73C14 is a low copy gene and may be a good candidate for genetic manipulation to improve fiber quality.

## Materials and Methods

### Chemicals

The majority of chemicals were purchased from Sigma-Aldrich (St. Louis, MO, USA). Indole 3 acetic acid (IAA), 1-naphthaleneacetic acid (NAA), 2,4-dichlorophenoacetic acid (2,4-D), and 6-benzylaminopurine (BAP) were obtained from PhytoTechnology Laboratories (Overland Park, KS, USA). Gossypol was extracted from cottonseed soapstock according to previously published procedure [43]. ABA-glucosyl ester, (+)-cis, trans-ABA and (+)-trans, trans- ABA were purchased from OlChemlm Ltd (Czech Republic); (+)-cis, trans-ABA and (-)-cis, trans-ABA were purchased from Sigma-Aldrich.

### Plant materials

The cotton short fiber mutant, *Li*_*2*_, was developed as a near-isogenic line (NIL) of the WT upland cotton line DP5690 as previously described [3]. Growth conditions, greenhouse experimental design, and fiber sampling were previously described [3]. Cotton bolls were harvested at the following time-points during development: -3 days before anthesis, day of anthesis (DOA), 1, 3, 5, 8, 12, 16, and 20 days post anthesis (DPA). Harvested bolls were placed on ice and transported to the laboratory and dissected. Roots and vegetative tissues, leaves, stems, and buds, were collected from greenhouse grown plants. Radicle, hypocotyl and cotyledon tissues were obtained from seedlings germinated for 5 days on wet filter paper. All tissues were frozen in liquid nitrogen and stored at -80°C.

### RNA isolation and RT-qPCR

Cotton fibers were isolated from developing ovules using a glass bead shearing technique to separate fibers from the ovules [44]. All other tissues were ground in liquid nitrogen and 100 mg of powder was weighed for RNA isolation. Total RNA was isolated using the Sigma Spectrum™ Plant Total RNA Kit (Sigma-Aldrich) with the optional on-column DNase1 digestion according to the manufacturer’s protocol. The concentration of each RNA sample was determined using a NanoDrop 2000 spectrophotometer (NanoDrop Technologies Inc., Wilmington, DE). The RNA quality for each sample was determined by RNA integrity number (RIN) using an Agilent Bioanalyzer 2100 and the RNA 6000 Nano Kit Chip (Agilent Technologies Inc., Santa Clara, CA). Quantitative RT-PCR was conducted as described before [3] according to the Minimum Information for Publication of Quantitative Real-Time PCR Experiments (MIQE) guidelines [45]. Normalization of RT-qPCR data was performed by geometric averaging three internal control genes, including 18S, UCP, and Tua4 [46]. The primer sequences and efficiencies are listed in Table S2.

### Affymetrix microarray and PSPG motif analysis

Affymetrix microarray analysis for samples at three developing time points of cotton fiber, DOA, 8 DPA and 12 DPA, representing initiation and peak of elongation stage, was previously published by our group [3]. Microarray data are available at ArrayExpress (http:www.ebi.ac.ud/arrayexpress, ID = E-MEXP-3306). The Affymetrix GeneChip ® Cotton Genome Array (Affymetrix Inc., Santa Clara, CA), contains 21,854 probe sets from four species of cotton ( *G* *. arboreum* , *G* *. barbadense* , *G. hirsutum*, and *G* *. raimondii* ). To identify sequences containing a UDP-binding consensus sequences, all probe sets were translated into the 6 possible reading frames (http://biotools.umassmed.edu/cgi-bin/biobin/transeq), then subjected to a Motif search using Geneious Pro software program [47] with the ExPASy Prosite accession #PS00375 for the PSPG motif [15,16].

### Cloning and construction of Arabidopsis expression vector

Two probe sets from Affymetrix microarray, Ghi.3235.1.A1_at and Ghi.9236.1.S1_at, represent truncated sequences of UGT. The 5’ and 3’ sequences were recovered by RACE-PCR using the BD SMART RACE cDNA amplification kit (BD Biosciences Clontech Inc., Palo Alto, CA) according to the manufacturer’s protocol. Gene specific primers used in RACE-PCR were: for Ghi.3235.1.A1_at GSP5’- 5’-tcatctcccagcatcgtaggcttgtt-3’ and GSP3’- 5’-aacggcaagcgaagtagacaagtgga-3’; for Ghi.9236.1.S1_at GSP5’ - 5’-agccacttcaaacacttctgcccatc-3’ and GSP3’- 5’-caccttacaatgcaggcagagtccaa-3’. The sequence was submitted to the UGT Nomenclature Committee for designation as UGT73C14 [15]. The full length cDNA sequence of UGT73C14 is available in GenBank, accession number JX846921. The PCR product of the open reading frame (ORF) of UGT73C14 was introduced into the entry vector using a pENTR/D-TOPO cloning kit (Invitrogen, Carlsbad, CA). The primer sequences used for directional cloning were 5’-caccatggctcaaggtcactttgtct-3’ and 5’-caacaatctcaggaaatgcgttag-3’. For *A. thaliana* transformation, the ORF of UGT73C14 was then transferred into binary vector pK2GW7 using GATEWAY conversion technology [48].

### *Arabidopsis thaliana* growth and transformation

*Arabidopsis thaliana* plants were grown in soil (Sunshine Aggregate Mix 4 Plus, Sun Gro Horticulture Distribution Inc., Bellevue, WA) in laboratory growth chambers (model AR66L-C8, Percival Scientific, Perry, IA) on a 16 h light/8 h dark regime at a light intensity of 150-165 µE/m^2^/sec. The UGT73C14 cDNA was inserted into a binary vector under the control of the constitutive CaMV 35S promoter and cloned into the *Agrobacterium tumefaciens* strain LBA4404 for Arabidopsis transformations. The *A. thaliana* ecotype Columbia was transformed using the floral dip method as previously described [49]. Seeds were collected from mature transformed T_0_ plants and stored in plastic tubes containing silica gel to reduce moisture. Transgenic T_1_ seeds were selected on plates containing solid media consisting of 2.2 g/L Murashige and Skoog basal salts, 2% sucrose, 50 µg/mL kanamycin, and 3 g/L phytagel, adjusted to pH 6.0 with dilute KOH solution. Kanamycin-resistant seedlings were transplanted to soil, and grown to maturity or used for tissue sampling and biochemical analysis.

### Copy Number Variation Assay

Leaves from *G. hirsutum* (wild type and *Li*_*2*_ mutant), *G* *. raimondii* , *G* *. herbaceum* and *G* *. arboreum* were collected, frozen and pulverized under liquid nitrogen, and stored at -80°C until further processing. Genomic DNA was isolated from the leaf tissue using Qiagen DNeasy Plant Mini Kit, catalog # 69104 (Qiagen, Germantown, Md), following the manufacturer’s instructions. High molecular weight DNA with no visible RNA contamination was confirmed by agarose gel electrophoresis. 23.84 ng of DNA (estimated to be 800 copies of tetraploid genome [50]) was subjected to a 5-cycle Specific Template Amplification (STA) reaction in a multiplex PCR using GhMYB25 and UGT73C14 primers (Table S2) to eliminate misinterpretation caused by potential tandem gene duplication [51] using Qiagen Multiplex PCR Kit, catalog #206143. Subsequent SYBR qPCR was conducted on genomic and STA reactions to verify that no bias in template was introduced during the STA reaction. The STA reaction for each sample was diluted 1:50 and 1 µL was added to a 10 µL MGB (Minor Groove Binding) Taqman reaction, set up according to the manufacturer’s instructions using catalog # PN4370048 (Applied Biosystems, Foster City, CA) for the reaction master mix and two technical replicates per sample. The probe sequence for GhMYB25 (EU826465.1) was 5’-VIC-CGCAATGCCTCGACGT-3’ and for UGT73C14 was 5’-FAM-ACTAGAGTCCAGTTGCC-3’. GhMYB25 was used as a reference gene as it has been previously demonstrated to be present in 1 copy for each genome [18]. The Taqman PCR mix was then loaded onto a primed 12.765 Digital Array, catalog # BMK-M-12.765 (Fluidigm Corporation, San Francisco, CA), and the mix was distributed to the 765 wells/reaction. After thermal cycling on the Fluidigm FC1 thermal cycler (95° for 10min, then 60 cycles of 95° for 15seconds and 60° for 1 minute), the microfluidic chip was analyzed on the EP1 chip reader (Fluidigm Corporation) to determine the number of wells containing a positive reaction. The ratio of positive wells for the unknown (UGT) to known (GhMYB25) reactions indicated the relative gene copy number. The copy number was calculated by multiplying the UGT73C14/GhMYB25 ratio by 4 for tetraploid species (*G. hirsutum*) and by 2 for diploid species ( *G* *. arboreum* , *G* *. herbaceum* , and *G* *. raimondii* ) [52]. Error bars indicate the 95% Confidence Interval.

### Cloning and expression of recombinant UTG73C14 in *E. coli*

The coding sequence for cotton UGT73C14 was fused at its N-terminus to the C-terminus of maltose-binding protein (MBP) via introduction into the *E. coli* expression vector pMAL-c5X. Aliquots of the primers GhUGT-Ala2Malc5 (5’-GCTCAAGGTCACTTTGTCTTGATC-3’) and GhUGT-3NotI (5’-ATCATGCGGCCGCTAACGCATTTCCTGAGATTGTTGG-3’) were treated with T4 polynucleotide kinase in 1X T4 DNA ligase buffer (containing 10 mM ATP, New England Biolabs, Ipswich, MA) at 37°C for 1 hour. The phosphorylated primer mix was used to amplify the open reading frame, minus the start methionine ATG codon, using Phusion High Fidelity DNA polymerase (New England Biolabs). The resulting PCR product was digested with *Not*I and ligated into pMAL-c5X that had been previously digested with *Xmn*I and *Not*I and treated with calf alkaline intestinal phosphatase. Ligation reactions were transformed into XL10-Gold Ultracompetent cells (Agilent Technologies) and selected on solid agar media containing 100 µg/mL ampicillin. Plasmid DNA was isolated from bacterial colonies bearing full-length inserts and sequenced to verify the reading frame and accuracy of the coding sequence.

Plasmids containing either the MBP-UGT fusion (pMBP-UGT73C14) or empty pMAL-c5x (a control plasmid for MBP expression) were transformed into BL21-CodonPlus (DE3)-RIPL competent cells and selected on solid agar media containing 50 µg/ml ampicillin and 34 µg/mL chloramphenicol. The optimum conditions for MBP-UTG73C14 expression were as follows: a single colony was inoculated into Luria-Bertani (LB)-Amp (100 μg/mL) medium (LB-Amp) and grown overnight with shaking at 37°C. The overnight culture was inoculated at a 1:20 dilution into fresh medium and grown for 2 h at 37°C to reach an optical cell density of approximately 0.6–1.0 at OD600nm. Isopropylthio-β-galactoside (IPTG) was added to the culture medium (0.5 mM final concentration) and protein expression was induced at 37°C for 2 h. Cells were collected by centrifugation at 10,000g for 10 min and homogenized by sonication in homogenization buffer (10 mL/L cultured cells) containing amylose resin wash buffer (20 mM Tris–HCl, pH 7.4, 200 mM NaCl, 10 mM β-mercaptoethanol, 1 mM EDTA), 0.2 mM phenylmethylsulfonyl fluoride (PMSF), and 1:500 dilution of protease inhibitor cocktail (Sigma-Aldrich, cat #P8340). The homogenate was centrifuged at 10,000g for 10 min to remove cell debris. The 10,000g supernatant was further centrifuged at 20,000g for 10 min.

### Purification of MBP-UGT73C14 with amylose resin affinity chromatography

The MBP-UGT73C14 was subjected to purification with an MBPTrap HP amylose resin affinity column, catalog # 28-9187-79 (GE Healthcare Life Sciences, Pittsburgh, PA), using fast protein liquid chromatography (FPLC) as described previously [53]. The 20,000g supernatant was loaded onto the MBPTrap HP column. The column was washed with 5 bed-volume of amylose resin wash buffer and then eluted with 20 bed-volume of a linear gradient with buffer A containing amylose resin wash buffer and buffer B containing amylose resin elution buffer (20 mM maltose in amylose resin wash buffer). A single protein peak was eluted between fraction 20 and fraction 22. The proteins in fractions 20-22 were separated by SDS-PAGE, detected by Coomassie brilliant blue staining, and identified as MBP-UGT73C14 by anti-MBP-mTTP antibodies (Figure S4).

### Protein determination, SDS–PAGE, and immunoblotting

Protein concentrations were determined with the Bradford method using the Protein Assay Dye Reagent Concentrate, catalog #500-0006 (Bio-Rad Laboratories, Hercules, CA) following 0.5 M NaOH treatment of the protein samples [54]. Proteins were separated by SDS–PAGE (4-20%) and visualized by staining with Coomassie brilliant blue R, catalog # B-7920 (Sigma-Aldrich). MBP-UGT was detected by immunoblotting following previously described procedures using nitrocellulose membranes and SuperSignal West Pico Chemiluminescent Substrate, catalog # 34079 (Pierce, Rockford, IL) [54]. The primary antibodies were rabbit anti-MBP-mTTP antibodies [55]. The secondary antibodies were affinity-purified goat anti-rabbit IgG (H+L) horseradish peroxidase conjugate (GAR-HRP), catalog #170-5046 (Bio-Rad Laboratories), with human IgG absorbed (Bio-Rad Laboratory) (Figure S4).

### Enzyme Assays

Enzyme reactions were performed with 5 µg of enzyme in a total volume of 100 µl containing 50 mM Tris-HCl pH 8.0, 50 mM MgCl_2_, 500 µM ATP, 2.5 mM UDP-glucose, UDP-galactose or UDP-glucuronic acid and 250 µM of substrate acceptor at 30°C for 2.5 hours. The negative controls had no enzyme. The reactions were stopped by filtering to remove enzyme using Millipore columns Cat #UFC503024 (Millipore, Billerica, MA) and analyzed by HPLC-MS.

For kinetic analysis of UGT73C14, purified enzyme (5 µg) was added to reaction mixture (100 µl final volume, in two technical replicates) containing 50 mM Tris-HCl pH 8.0, 50 mM MgCl_2_, 500 µM ATP, 2.5 mM UDP-glucose or UDP-galactose and 0-125 µM acceptor substrate. Reactions were stopped with 4 volumes of acetone after 1 hour incubation at 30°C; protein was removed by precipitation for 1hour at -20°C and centrifugation for 10 min at 14000 g; acetone was evaporated from supernatant in SpeedVac for 40 min; aqueous fractions were analyzed by HPLC-MS. Standard curve of ABA glucosyl ester (OlChemlm Ltd) was used to calculate amount of product. Data were analyzed using Hyper32 software (http://homepage.ntlworld.com/john.easterby/hyper32.html); the kinetic parameters were determined by Hyperbolic Regression Analysis.

### HPLC-mass spectrometry analysis of in vitro assay

High performance liquid chromatography – mass spectrometry (HPLC-MS) analyses were carried out with an Alliance 2695 HPLC, with column heater, 996 Photodiode Array Detector, and LCT Premier XE time-of-flight mass spectrometer (Waters, Milford, MA). A Luna C18, 3 µm, 2.0 x 50 mm column (Phenomenex, Torrance, CA) was used at 30°C. The MS was equipped with an electrospray ionization (ESI) source and parameters set as: capillary voltage, 3000 V (ES+), 2600 V (ES-); cone voltage, 30 V; desolvation heater, 450°C; source temp, 120°C; desolvation gas, 500 L/hr; cone gas flow, 50 L/hr; V mode (resolution = 6500), positive and negative ionization polarity, scan range m/z 130-1000. Leu-enkephalin was used for the lock spray. A 20 μl aqueous sample was injected onto the column and eluted with 0.5% acetonitrile in 0.1% formic acid (aq.) for 10 min followed by a gradient from 0.5% to 70% acetonitrile in 20 min, a 5 min hold, and a 70% to 99% gradient in 0.1 min, at a flow rate of 0.3 ml/min.

### UGT73C14 transformed *A. thaliana* growth in Abscisic Acid

The post-germinative growth experiment was performed as described before [39]. T2 transgenic *A. thaliana* seeds (10mg/line) were surface sterilized in 1.5ml 10% bleach with 0.1% SDS by gentle shaking for 15 minutes, rinsed with sterile water and maintained at 4°C overnight. Seeds were then plated on 2.5g/L Phytagel supplemented with 2.2g/L Murashige and Skoog basal salts at pH 6.0. Each transgenic or wild type line was plated with and without filter sterilized 0.5μM Abscisic Acid (Sigma-Aldrich) in the phytagel. Immediately upon plating the number of seeds per plate was counted (approximately 100 seeds/plate), with two plates per experimental condition. After 4 days at 4°C in darkness, germination occurred over 14 days in 8: 16 hr dark: light cycles at 18°C and 22°C, respectively. The number of germinated seedlings showing 2 emerged cotyledons was counted on days 3, 7, 10, and 14. Two-Way ANOVA (GraphPad Prism 5 software) was utilized for statistical analysis of data using variables: transgenic effect and time (Data S1).

## Supporting Information

Table S1 **Accession numbers, references and details regarding substrate acceptors, donors and products for the UGTs used in phylogenetic analysis (Figure 3).**
(DOCX)

Table S2 **Primer sequences and efficiency test results for primers used in qPCR analysis.**
(DOCX)

Figure S1 **Sequence Alignment of PSPG motif found in Affymetrix microarray probes.** Consensus sequences of probe sets were translated into the 6 possible reading frames (http://biotools.umassmed.edu/cgi-bin/biobin/transeq), then subjected to a Motif search using Geneious Pro software program [47] with the ExPASy Prosite accession #PS00375 for the plant secondary product glycosyltransferase (PSPG) motif [15,16].
(TIF)

Figure S2 Transcript patterns of UGTs. A correlation analysis of transcript values was performed with a Prism 5 software (www.graphpad.com) using Pearson method with confidence interval 95% (two tailed). The correlation coefficient (r) is shown on graph from right. Abbreviations: S, stem; R, radicle; H, hypocotyls; C, cotyledon; L, leaf; B, bud; Rt, root. Enriched epidermal cell fraction of ovules at -3, -1 days before anthesis and 0 day of anthesis; fiber cells at 1, 3, 5, 8, 12, 16 and 20 days after anthesis.
(TIF)

Figure S3 Amino acid alignment of UGT73C14 and functionally characterized UGTs used for phylogenetic analysis. The amino acid sequences were aligned using MUSCLE program [58]. Visualization of alignment was performed using protein boxshade generator (http://www.fr33.net/boxshadeprotein.php). Glutamine (Q) and histidine (H) amino acids at the C-terminal of PSPG box indicated by arrow are highly conserved among glucosyltransferases and galactosyltransferases, respectively.
(DOCX)

Figure S4 FPLC purification of MBP-UGT73C14. The MBP-UGT73C14 was subjected to purification with an amylose resin affinity column using fast protein liquid chromatography (FPLC). A single protein peak was eluted between fractions 20-22. The proteins in fractions 20-22 were separated by SDS-PAGE, detected by Coomassie brilliant blue staining (CBB), and identified as MBP-UGT73C14 by anti-MBP-mTTP antibodies using Western Blotting (WB) (inset). The purified MBP-UGT73C14 contained partially degraded fragments of the recombinant protein with molecular weight about 99 kDa.
(TIF)

Figure S5 Substrates tested to determine UGT73C14 activity. The indicated substrates were tested individually in *in* *vitro* enzymatic reactions with 5 µg of enzyme in a total volume of 100 µl containing 50 mM Tris-HCl pH 8.0, 50 mM MgCl_2_, 500 µM ATP, 2.5 mM UDP-glucose, UDP-galactose or UDP-glucuronic acid and 250 µM of substrate acceptor at 30°C for 2.5 hours. The negative controls had no enzyme. Substrate that showed activity with UGT73C14 is highlighted with a grey filling.
(TIF)

Data S1 Statistical analysis of cotyledon emergence rate between transgenic and wild type plants. (TXT)
