# Supplementary material for: A Transcript Profiling Approach Reveals an Abscisic Acid-Specific Glycosyltransferase (UGT73C14) Induced in Developing Fiber of Ligon lintless-2 Mutant of Cotton (Gossypium hirsutum L.)
Source: PLoS One. 2013 Sep 23;8(9):e75268. doi: 10.1371/journal.pone.0075268 (PMC3781043; doi:10.1371/journal.pone.0075268)
Supplement: Table S1 — Accession numbers, references and details regarding substrate acceptors, donors and products for the UGTs used in phylogenetic analysis (Figure 3). (DOCX) [file pone.0075268.s001.docx]

| Nomencl. name | Alterna-tive name | Species | GenBank no. | Major aglycone acceptor | Sugar donor | Products | References |
| --- | --- | --- | --- | --- | --- | --- | --- |
| UGT71A9 |  | *Sesamum indicum* | BAF96582 | Lignan:  (+)-Sesaminol | UDPGlc | (+)-Sesaminol 2-O-β-D-Glc | [[1](#_ENREF_1)] |
| UGT71B6 |  | *Arabidopsis thaliana* | NP_188815 | (+)-ABA | UDPGlc | (+)-ABA-Glc | [[2](#_ENREF_2)] |
| UGT71G1 | GT29H | *Medicago truncatula* | AAW56092 | (Iso)Flavonoids,  Triterpenes: (Hederagenin) | UDPGlc | Multiple products of quercetin; Isoflavonoid 7-O-Glc; Hederagenin 3- or 28-O-Clc | [[3](#_ENREF_3),[4](#_ENREF_4)] |
| UGT72B1 |  | *Arabidopsis thaliana* | 2VCE_A | Chloroanilines | UDPXyl  UDPGlc | Chloroanilines-O- /or -N-Glc | [[5](#_ENREF_5)] |
| UGT72L1 |  | *Medicago truncatula* | ACC38470 | (-)-Epicatechin | UDPGlc | Epicatechin 3'-O-Glc | [[6](#_ENREF_6)] |
|  | ZOG1 | *Phaseolus lunatus* | AAD04166 | Zeatin | UDPGlc | Zeatin-O-Glc | [[7](#_ENREF_7)] |
|  | ZOX1 | *Phaseolus vulgaris* | AAD51778 | Zeatin | UDPXyl | Zeatin-O-Xyl | [[7](#_ENREF_7)] |
| UGT73A1 | TOGT1 | *Nicotina tabacum* | AAB36653 | Coumarins | UDPGlc | Scopolin, esculin | [[8](#_ENREF_8)] |
| UGT73B1 | AtF7GT | *Arabidopsis thaliana* | AAL90934 | Flavonoids | UDPGlc | Flavonoid 7-O-Glc | [[9](#_ENREF_9)] |
|  | B5GT | *Dorotheanthus bellidiformis* | CAB56231 | Betanidin, Flavonoids | UDPGlc | Betanidin-5-O-Glc | [[10](#_ENREF_10)] |
| UGT73C1 |  | *Arabidopsis thaliana* | Q9ZQ99 | Zeatin | UDPGlc | Zeatin-O-Glc | [[11](#_ENREF_11)] |
| UGT73C5 |  | *Arabidopsis thaliana* | Q9ZQ94 | Zeatin,  brassinolide | UDPGlc | Zeatin-O-Glc  BL-23-O-Glc | [[11](#_ENREF_11),[12](#_ENREF_12)] |
| UGT73C6 | AtF3G7GT | *Arabidopsis thaliana* | Q9ZQ95 | Flavonol-3-O-Glc | UDPGlc | Flavonol-3-O-Glc-7-O-Glc | [[13](#_ENREF_13)] |
| **UGT73C14** |  | *Gossypium hirsutum* | JX846921 | ABA | UDPGlu  UDPGal | ABA-Glu, ABA-Gal | This study |
| UGT73C8 | GT22D | *Medicago truncatula* | ABI94020 | (Iso)Flavonoids, | UDPGlc | (Iso)Flavonoid 7-O-Glc | [[4](#_ENREF_4)] |
| UGT73F3 | GT3 | *Medicago truncatula* | ACT34898 | Triterpenes:  (Hederagenin) | UDPGlc | Hederagenin 28-O-Clc | [[14](#_ENREF_14)] |
| UGT73K1 | GT49F | *Medicago truncatula* | AAW56091 | Triterpenes:  (Hederagenin) | UDPGlc | Hederagenin 3- or 28-O-Clc | [[3](#_ENREF_3)] |
| UGT73P2 | GmSGT2 | *Glycine max* | BAI99584 | Soyasapogenol B monoglucuronide | UDPGal | Soyasaponin III | [[15](#_ENREF_15)] |
| UGT75C1 | AtF5GT | *Arabidopsis thaliana* | AAM91686 | Flavonoids | UDPGlc | Flavonoid 5-O-Glc | [[16](#_ENREF_16)] |
|  | AOG | *Vigna angularis* | Q8W3P8 | (+)-ABA | UDPGlc | ABA-Glc | [[17](#_ENREF_17)] |
|  | A5GT | *Perilla frutescens* | BAA36421 | Anthocyanidin-3-O-Glc | UDPGlc | Anthocyanidin-3,5-O-di-Glc | [[18](#_ENREF_18)] |
|  | iaglu | *Zea mays* | AAA59054 | IAA | UDPGlc | 1-O-1A-Glc | [[19](#_ENREF_19)] |
| UGT76C1 |  | *Arabidopsis thaliana* | AED90934 | Zeatin, kinetin,  IPA, BAP | UDPGlc | Cytokinin-N-Glc | [[11](#_ENREF_11)] |
| UGT76C2 |  | *Arabidopsis thaliana* | AED90933 | Zeatin, kinetin,  IPA, BAP | UDPGlc | Cytokinin-N-Glc | [[11](#_ENREF_11)] |
|  | GhUGT1 | *Gossypium hirsutum* | ABN58740 | NA | NA | NA | [[20](#_ENREF_20)] |
| UGT78B1 | UF3GT | *Gentiana triflora* | BAA12737 | Anthocynidins, Flavonols | UDPGlc | Anthocyanidin (Flavonol)-3-O-Glc | [[21](#_ENREF_21)] |
|  | F3GalT | *Petunia hybrida* | AAD55985 | Flavonols | UDPGal | Flavonol-3-O-Gal | [[22](#_ENREF_22)] |
|  | AcGaT | *Aralia cordata* | BAD06514 | Flavonoids | UDPGal | Flavonoid 3-O-Gal | [[23](#_ENREF_23)] |
| UGT78D2 | AtF3GT | *Arabidopsis thaliana* | AAM91139 | Flavonoids | UDPGlc | Flavonoid 3-O-Glc | [[16](#_ENREF_16)] |
|  | UF3GT | *Vitis vinifera* | AAB81683 | Anthocynidins, Flavonols | UDPGlc | Anthocyanidin (Flavonol)-3-O-Glc | [[24](#_ENREF_24)] |
| UGT78G1 | GT83F | *Medicago truncatula* | ABI94025 | (Iso)Flavonoids, | UDPGlc | (Iso)Flavonoid 7-O-Glc; Flavonoid 3-O-Glc | [[4](#_ENREF_4)] |
| UGT85A1 |  | *Arabidopsis thaliana* | AEE30237 | Zeatin | UDPGlc | Zeatin-O-Glc | [[11](#_ENREF_11)] |
| UGT85H2 | GT67A | *Medicago truncatula* | ABI94024 | (Iso)Flavonoids, | UDPGlc | (Iso)Flavonoid 7-O-Glc; Flavonoid 3-O-Glc | [[4](#_ENREF_4)] |
|  | HMNGT | *Sorghum bicolor* | AAF17077 | Hydroxymandclo-nitrile | UDPGlc | Dhurrin | [[25](#_ENREF_25)] |
| UGT88E1 | GT22E09 | *Medicago truncatula* | ABI94021 | (Iso)Flavonoids, | UDPGlc | (Iso)Flavonoid 7-O-Glc | [[4](#_ENREF_4)] |
| UGT88E2 | GT29C | *Medicago truncatula* | ABI94022 | (Iso)Flavonoids, | UDPGlc | (Iso)Flavonoid 7-O-Glc | [[4](#_ENREF_4)] |
| UGT91H4 | GmSGT3 | *Glycine max* | BAI99585 | Soyasaponin III | UDPRha | Soyasaponin I | [[15](#_ENREF_15)] |
|  | 3RT | *Petunia hybrida* | CAA50376 | Anthocyanidin-3-O-Glc | UDPRha | Anthocyanidin-3-O-Glc 6’-Rha | [[26](#_ENREF_26)] |
| UGT94B1 | BpUGAT | *Bellis perennis* | BAD77944 | Cyanidin 3-O-Glc | UDPGlcA | cyanidin 3-O-β-2”-O-β-glucuronosyl-6”-O-malonylglucopyranoside | [[27](#_ENREF_27)] |
|  | CmFG2RT | *Citrus maxima* | AAL06646 | Flavanone 7-O-Glc | UDPRha | Flavanone-7-O-neohesperidoside | [[28](#_ENREF_28)] |
| UGT94D1 |  | *Sesamum indicum* | BAF99027 | Lignan: (+)-Sesaminol 2-O-β-D-Glc | UDPGlc | Sesaminol 2-O-β-D-Glc (1-6)-O-β-D-Glc | [[1](#_ENREF_1)] |

# References:

1. Noguchi A, Fukui Y, Iuchi-Okada A, Kakutani S, Satake H, et al. (2008) Sequential glucosylation of a furofuran lignan, (+)-sesaminol, by *Sesamum indicum* UGT71A9 and UGT94D1 glucosyltransferases. Plant J 54: 415-427.

2. Priest DM, Jackson RG, Ashford DA, Abrams SR, Bowles DJ (2005) The use of abscisic acid analogues to analyse the substrate selectivity of UGT71B6, a UDP-glycosyltransferase of *Arabidopsis thaliana*. FEBS Lett 579: 4454-4458.

3. Achnine L, Huhman DV, Farag MA, Sumner LW, Blount JW, et al. (2005) Genomics-based selection and functional characterization of triterpene glycosyltransferases from the model legume *Medicago truncatula*. Plant J 41: 875-887.

4. Modolo LV, Blount JW, Achnine L, Naoumkina MA, Wang X, et al. (2007) A functional genomics approach to (iso)flavonoid glycosylation in the model legume *Medicago truncatula*. Plant Mol Biol 64: 499-518.

5. Brazier-Hicks M, Offen WA, Gershater MC, Revett TJ, Lim E-K, et al. (2007) Characterization and engineering of the bifunctional N- and O-glucosyltransferase involved in xenobiotic metabolism in plants. Proc Natl Acad Sci U S A 104: 20238-20243.

6. Pang Y, Peel GJ, Sharma SB, Tang Y, Dixon RA (2008) A transcript profiling approach reveals an epicatechin-specific glucosyltransferase expressed in the seed coat of *Medicago truncatula*. Proc Natl Acad Sci U S A 105: 14210-14215.

7. Martin RC, Mok MC, Mok DW (1999) A gene encoding the cytokinin enzyme zeatin O-xylosyltransferase of *Phaseolus vulgaris*. Plant physiology 120: 553-558.

8. Fraissinet-Tachet L, Baltz R, Chong J, Kauffmann S, Fritig B, et al. (1998) Two tobacco genes induced by infection, elicitor and salicylic acid encode glucosyltransferases acting on phenylpropanoids and benzoic acid derivatives, including salicylic acid. FEBS Lett 437: 319-323.

9. Kim JH, Kim BG, Park Y, Ko JH, Lim CE, et al. (2006) Characterization of flavonoid 7-O-glucosyltransferase from *Arabidopsis thaliana*. Biosci Biotechnol Biochem 70: 1471-1477.

10. Vogt T, Grimm R, Strack D (1999) Cloning and expression of a cDNA encoding betanidin 5-O-glucosyltransferase, a betanidin- and flavonoid-specific enzyme with high homology to inducible glucosyltransferases from the Solanaceae. Plant J 19: 509-519.

11. Hou B, Lim E-K, Higgins GS, Bowles DJ (2004) N-Glucosylation of Cytokinins by Glycosyltransferases of *Arabidopsis thaliana*. J Biol Chem 279: 47822-47832.

12. Poppenberger B, Fujioka S, Soeno K, George GL, Vaistij FE, et al. (2005) The UGT73C5 of *Arabidopsis thaliana* glucosylates brassinosteroids. Proc Natl Acad Sci U S A 102: 15253-15258.

13. Jones P, Messner B, Nakajima J, Schaffner AR, Saito K (2003) UGT73C6 and UGT78D1, glycosyltransferases involved in flavonol glycoside biosynthesis in *Arabidopsis thaliana*. J Biol Chem 278: 43910-43918.

14. Naoumkina MA, Modolo LV, Huhman DV, Urbanczyk-Wochniak E, Tang Y, et al. (2010) Genomic and coexpression analyses predict multiple genes involved in triterpene saponin biosynthesis in *Medicago truncatula*. Plant cell 22: 850-866.

15. Shibuya M, Nishimura K, Yasuyama N, Ebizuka Y (2010) Identification and characterization of glycosyltransferases involved in the biosynthesis of soyasaponin I in *Glycine max*. FEBS Lett 584: 2258-2264.

16. Tohge T, Nishiyama Y, Hirai MY, Yano M, Nakajima J, et al. (2005) Functional genomics by integrated analysis of metabolome and transcriptome of Arabidopsis plants over-expressing an MYB transcription factor. Plant J 42: 218-235.

17. Xu ZJ, Nakajima M, Suzuki Y, Yamaguchi I (2002) Cloning and characterization of the abscisic acid-specific glucosyltransferase gene from adzuki bean seedlings. Plant Physiol 129: 1285-1295.

18. Yamazaki M, Gong Z, Fukuchi-Mizutani M, Fukui Y, Tanaka Y, et al. (1999) Molecular cloning and biochemical characterization of a novel anthocyanin 5-O-glucosyltransferase by mRNA differential display for plant forms regarding anthocyanin. J Biol Chem 274: 7405-7411.

19. Szerszen JB, Szczyglowski K, Bandurski RS (1994) *iaglu*, a gene from *Zea mays* involved in conjugation of growth hormone indole-3-acetic acid. Science 265: 1699-1701.

20. Tai F, Wang X, Xu W, Li X (2008) Characterization and expression analysis of two cotton genes encoding putative UDP-Glycosyltransferases. Mol Biol 42: 44-51.

21. Tanaka Y, Yonekura K, Fukuchi-Mizutani M, Fukui Y, Fujiwara H, et al. (1996) Molecular and biochemical characterization of three anthocyanin synthetic enzymes from *Gentiana triflora*. Plant Cell Physiol 37: 711-716.

22. Miller KD, Guyon V, Evans JN, Shuttleworth WA, Taylor LP (1999) Purification, cloning, and heterologous expression of a catalytically efficient flavonol 3-O-galactosyltransferase expressed in the male gametophyte of *Petunia hybrida*. J Biol Chem 274: 34011-34019.

23. Kubo A, Arai Y, Nagashima S, Yoshikawa T (2004) Alteration of sugar donor specificities of plant glycosyltransferases by a single point mutation. Arch Biochem Biophys 429: 198-203.

24. Ford CM, Boss PK, Hoj PB (1998) Cloning and characterization of *Vitis vinifera* UDP-glucose:flavonoid 3-O-glucosyltransferase, a homologue of the enzyme encoded by the maize Bronze-1 locus that may primarily serve to glucosylate anthocyanidins in vivo. J Biol Chem 273: 9224-9233.

25. Jones PR, Moller BL, Hoj PB (1999) The UDP-glucose:p-hydroxymandelonitrile-O-glucosyltransferase that catalyzes the last step in synthesis of the cyanogenic glucoside dhurrin in *Sorghum bicolor*. Isolation, cloning, heterologous expression, and substrate specificity. J Biol Chem 274: 35483-35491.

26. Kroon J, Souer E, de Graaff A, Xue Y, Mol J, et al. (1994) Cloning and structural analysis of the anthocyanin pigmentation locus *Rt* of *Petunia hybrida*: characterization of insertion sequences in two mutant alleles. The Plant Journal 5: 69-80.

27. Sawada S, Suzuki H, Ichimaida F, Yamaguchi MA, Iwashita T, et al. (2005) UDP-glucuronic acid:anthocyanin glucuronosyltransferase from red daisy (*Bellis perennis*) flowers. Enzymology and phylogenetics of a novel glucuronosyltransferase involved in flower pigment biosynthesis. J Biol Chem 280: 899-906.

28. Frydman A, Weisshaus O, Bar-Peled M, Huhman DV, Sumner LW, et al. (2004) Citrus fruit bitter flavors: isolation and functional characterization of the gene Cm1,2RhaT encoding a 1,2 rhamnosyltransferase, a key enzyme in the biosynthesis of the bitter flavonoids of citrus. Plant J 40: 88-100.
