# Supplementary material for: A Transcript Profiling Approach Reveals an Abscisic Acid-Specific Glycosyltransferase (UGT73C14) Induced in Developing Fiber of Ligon lintless-2 Mutant of Cotton (Gossypium hirsutum L.)
Source: PLoS One. 2013 Sep 23;8(9):e75268. doi: 10.1371/journal.pone.0075268 (PMC3781043; doi:10.1371/journal.pone.0075268)
Supplement: Table S2 — Primer sequences and efficiency test results for primers used in qPCR analysis. (DOCX) [file pone.0075268.s002.docx]

| **Name** | **Forward primer sequence, 5’-3’** | **Reverse primer sequence, 5’-3’** | **Eff, %** |
| --- | --- | --- | --- |
| Ghi.3235.1.A1_at | GGGCTAACAAGCCTACGATG | TCGCCTCATCTCCTAATTCTTT | 97.5 |
| Ghi.9236.1.S1_at | AGCTGACCGAGCATCATATGGAGT | TCTTTGTGGCTGAGAGACACTGGA | 85.1 |
| GhiAffx.22326.1.A1_s_at | AGGAAACGGTATCCGAAAGAGAGC | AACGGCTTGTAGAACCCTCTTCCA | 84.2 |
| GhiAffx.53295.1.A1_at | ATGAAGATTGCGTTGCCGATGGTG | GCTGCATGTTTCATGGCTAAGGCT | 94.0 |
| Ghi.6369.2.A1_at | AGCAAATGCTATCTAACAGGGATCT | GGCAGAACATTACATGCAAGAACACC | 83.9 |
| GhiAffx.10836.1.A1_at | GAGGTGGCGAAATGCTTTGTGGAA | ACGTGATTCATTGCCATGCTCGTG | 87.2 |
| Ghi.10155.1.S1_at | TAAACGGTAAGATGGGACACCCGA | TGCCCACCCGATGATCTTACCAAT | 82.4 |
| GraAffx.29373.1.A1_at | AACTCACATTCCTCCAGGGCTTCA | TCCTCAACGTTGGTCCCTTCACTT | 90.1 |
| Ghi.5701.1.A1_at | TCCACGATTGAAAGCATATCCGGC | AGCTCTCTAACACGGCTCTCTACT | 80.1 |
| Ghi.8601.1.S1_s_at | ACCCGAGTTGCAGGCTTAGTTCTT | TCGGGTACTCAAATTCCGAGCTGA | 84.8 |
| GraAffx.22950.1.A1_s_at | AGGAGGTGGGATTAGCAGTAGAGT | TCCACCATTCATAACGGCCTTCCT | 92.0 |
| GhiAffx.6353.1.A1_s_at | TCCTGCACCCATTCCGATTGATGA | TGCAACTCCATCCTCACCTTCCAT | 81.1 |
| GhiAffx.6503.1.A1_at | GGTGTTTCGTGACTCATTGTGGCT | TCCAATTCTCAAGTCTCCGGCCAT | 84.6 |
| GhZEP | AGCCCCTTCTTTTGGTTGTT | TCAGTTTCCAGTTTCCGTTTC | 93.1 |
| GhNSY | ATCCTGAAAAACCTGCCTTG | GTCATGGACGCCTGATACATT | 89.6 |
| GhNCED | GGAAGAACAAAGGCTCACCA | GGAAGAACAAAGGCTCACCA | 92.5 |
| GhSDR | CGAGCTTTCCGTACTTGGAG | TGCACAGCCACACTTTTTGT | 98.3 |
| 18S | CGTCCCTGCCCTTTGTACA | AACACTTCACCGGACCATTCA | 94.4 |
| UCP | CGGAAAGAGGTGAAGATGTCAAC | GGATCTTGCTGCAACCTCTTAAA | 81.6 |
| Tua4 | GATCTCGCTGCCCTGGAA | ACCAGACTCAGCGCCAACTT | 89.8 |
| UGT (taqman) | GGTTTGACCGACAAGGTG | CCTTCCAGGATCCATCATTGTC | 97.3 |
| Myb25 (taqman) | CCGGCAACTAGACCGCAAT | AACCCGCAGACGACACCTT | 100.5 |
