# Supplementary figures and images for: A Transcript Profiling Approach Reveals an Abscisic Acid-Specific Glycosyltransferase (UGT73C14) Induced in Developing Fiber of Ligon lintless-2 Mutant of Cotton (Gossypium hirsutum L.)
Source: PLoS One. 2013 Sep 23;8(9):e75268. doi: 10.1371/journal.pone.0075268 (PMC3781043; doi:10.1371/journal.pone.0075268)

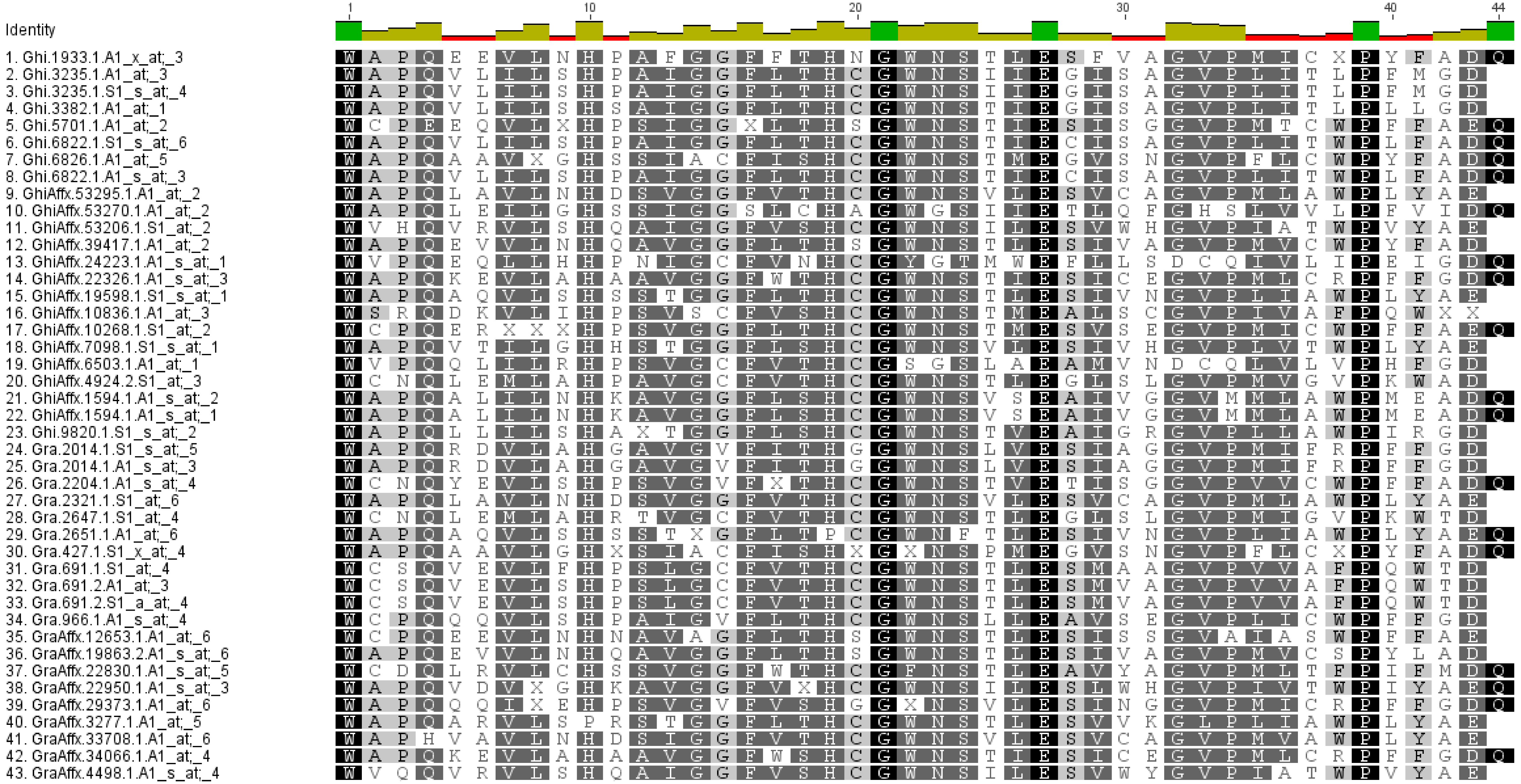

Supplement: Figure S1 — Sequence Alignment of PSPG motif found in Affymetrix microarray probes. Consensus sequences of probe sets were translated into the 6 possible reading frames (http://biotools.umassmed.edu/cgi-bin/biobin/transeq), then subjected to a Motif search using Geneious Pro software program [47] with the ExPASy Prosite accession #PS00375 for the plant secondary product glycosyltransferase (PSPG) motif [15,16]. (TIF) [file pone.0075268.s003.tif]

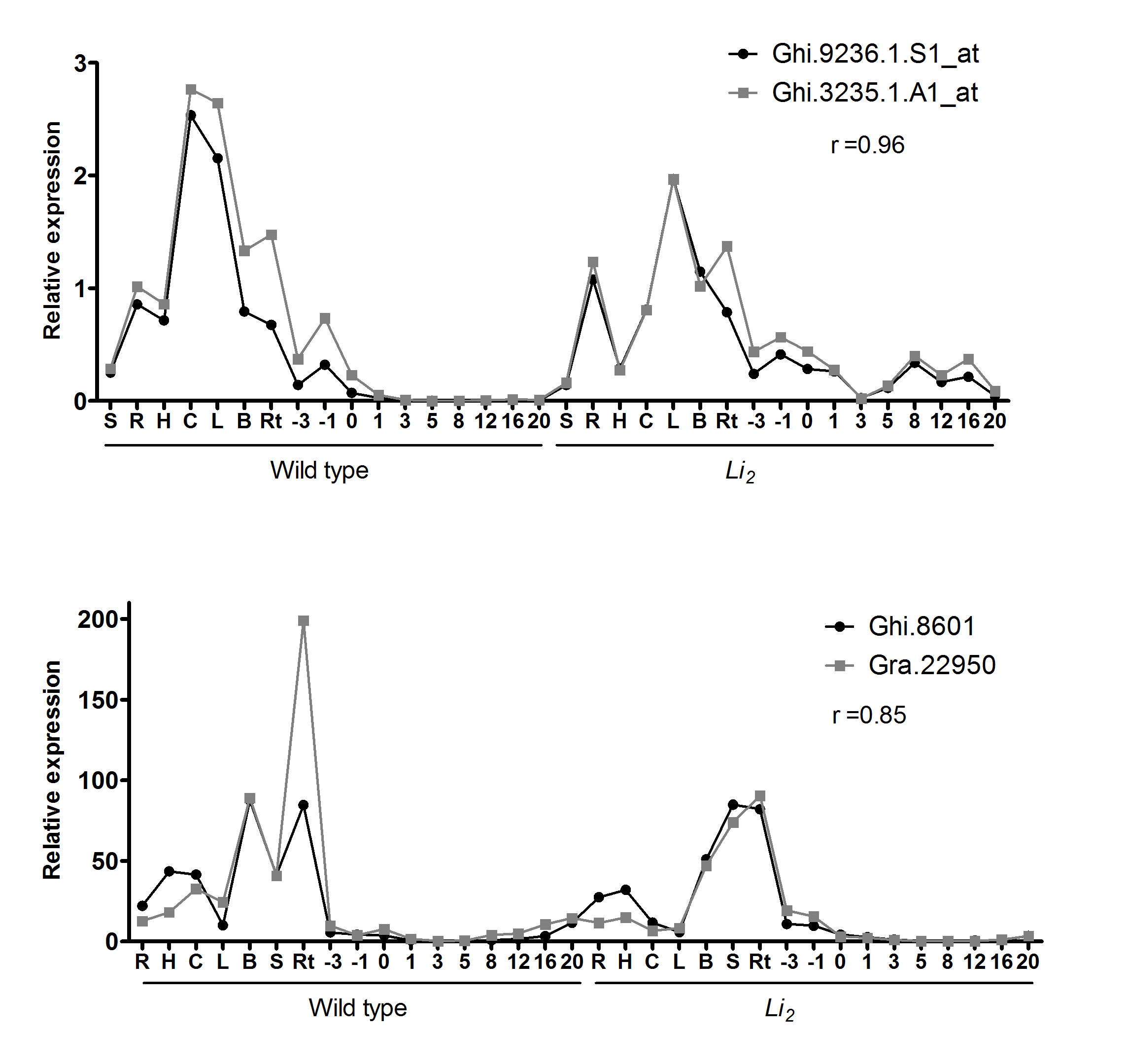

Supplement: Figure S2 — A correlation analysis of transcript values was performed with a Prism 5 software (www.graphpad.com) using Pearson method with confidence interval 95% (two tailed). The correlation coefficient (r) is shown on graph from right. Abbreviations: S, stem; R, radicle; H, hypocotyls; C, cotyledon; L, leaf; B, bud; Rt, root. Enriched epidermal cell fraction of ovules at -3, -1 days before anthesis and 0 day of anthesis; fiber cells at 1, 3, 5, 8, 12, 16 and 20 days after anthesis. (TIF) [file pone.0075268.s004.tif]

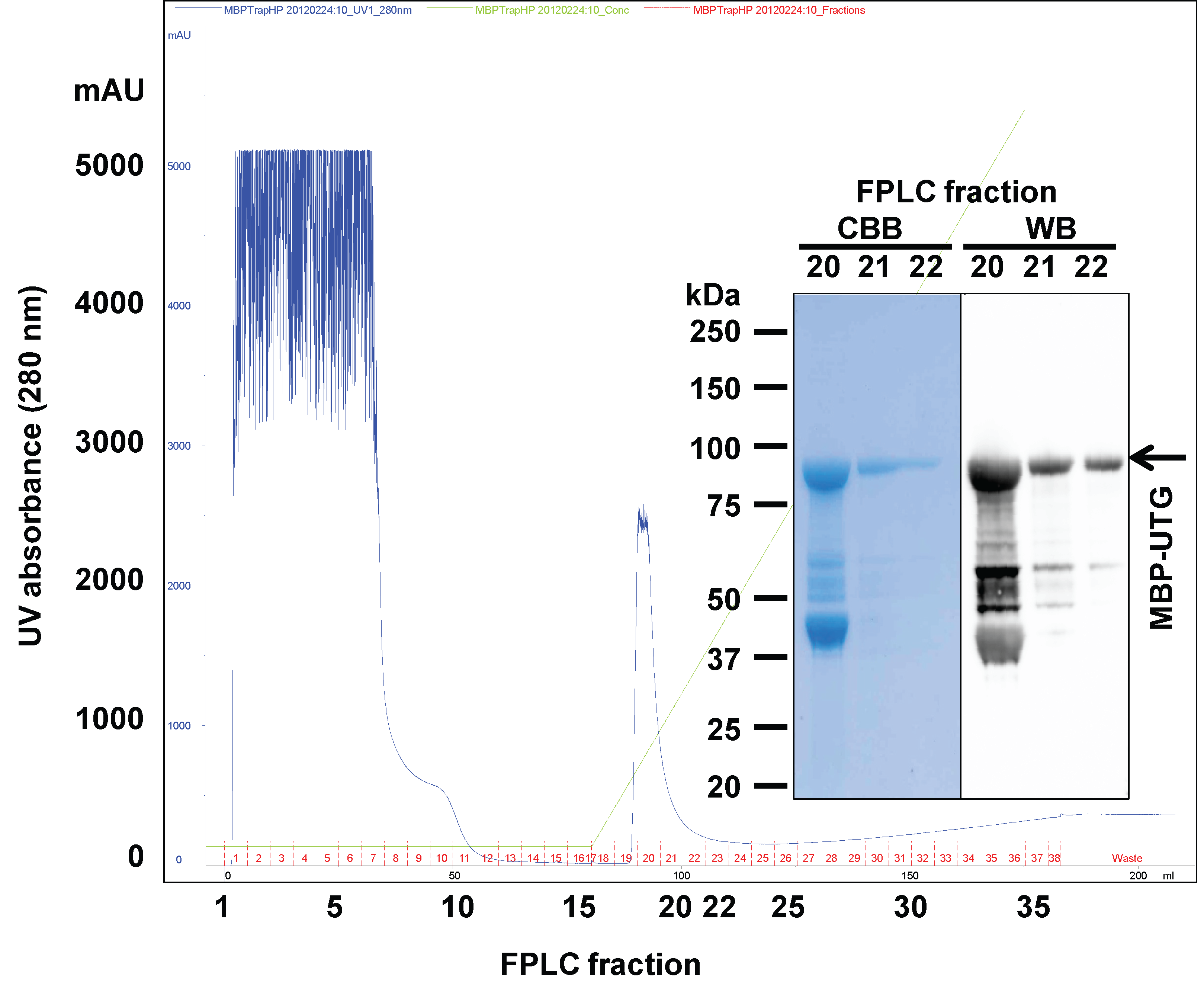

Supplement: Figure S4 — The MBP-UGT73C14 was subjected to purification with an amylose resin affinity column using fast protein liquid chromatography (FPLC). A single protein peak was eluted between fractions 20-22. The proteins in fractions 20-22 were separated by SDS-PAGE, detected by Coomassie brilliant blue staining (CBB), and identified as MBP-UGT73C14 by anti-MBP-mTTP antibodies using Western Blotting (WB) (inset). The purified MBP-UGT73C14 contained partially degraded fragments of the recombinant protein with molecular weight about 99 kDa. (TIF) [file pone.0075268.s006.tif]

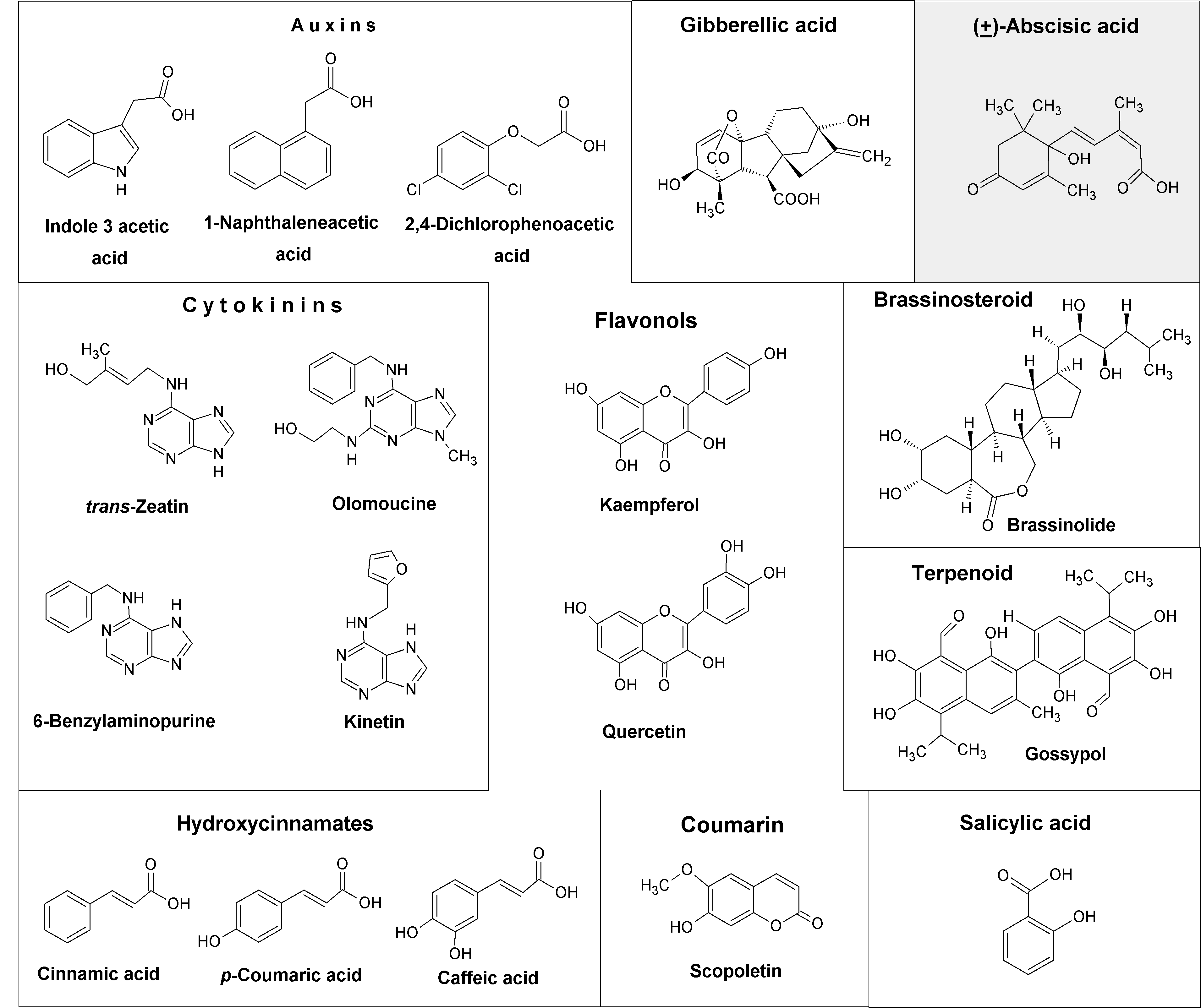

Supplement: Figure S5 — The indicated substrates were tested individually in in vitro enzymatic reactions with 5 µg of enzyme in a total volume of 100 µl containing 50 mM Tris-HCl pH 8.0, 50 mM MgCl2, 500 µM ATP, 2.5 mM UDP-glucose, UDP-galactose or UDP-glucuronic acid and 250 µM of substrate acceptor at 30°C for 2.5 hours. The negative controls had no enzyme. Substrate that showed activity with UGT73C14 is highlighted with a grey filling. (TIF) [file pone.0075268.s007.tif]
