# Supplementary material for: A Transcript Profiling Approach Reveals an Abscisic Acid-Specific Glycosyltransferase (UGT73C14) Induced in Developing Fiber of Ligon lintless-2 Mutant of Cotton (Gossypium hirsutum L.)
Source: PLoS One. 2013 Sep 23;8(9):e75268. doi: 10.1371/journal.pone.0075268 (PMC3781043; doi:10.1371/journal.pone.0075268)
Supplement: Figure S3 — The amino acid sequences were aligned using MUSCLE program [58]. Visualization of alignment was performed using protein boxshade generator (http://www.fr33.net/boxshadeprotein.php). Glutamine (Q) and histidine (H) amino acids at the C-terminal of PSPG box indicated by arrow are highly conserved among glucosyltransferases and galactosyltransferases, respectively. (DOCX) [file pone.0075268.s005.docx]

(100.0%) GhUGT73C14 1 -----------MAQGHFVLIPFMAQGHLIPMVDIGRLL--AQRGVTVT-IVTTPYNAGRV
(23.2%) Zm_iaglu 1 ------------MAPHVLVVPFPGQGHMNPMVQFAKRL--ASKGVATT-LVTTRFIQRTA
(22.0%) Gtr_UGT78B1 1 ----------MSPVSHVAVLAFPFGTHAAPLLTLVNRLAASAPDIIFS-FFSTSSSITTI
(40.8%) Nt_UGT73A1 1 -----------MGQLHIFFFPVMAHGHMIPTLDMAKLF--ASRGVKAT-IITTPLNEFVF
(25.7%) Pv_ZOX1 1 ------MALNDETKVVVLLLPFPVQGHLNPFLQLSHLI--AAQNIAVH-YVGTVTHIRQA
(22.5%) Pf_A5GT 1 -----------MVRRRVLLATFPAQGHINPALQFAKRL--LKAGTDVT-FFTSVYAWRRM
(23.2%) Sb_HMNGT 1 ----MGSNAPPPPTPHVVLVPFPGQGHVAPLMQLARLL--HARGARVT-FVYTQYNYRRL
(22.9%) Ph_F3GalT 1 -----------MSNYHVAVLAFPFATHAGLLLGLVQRLANALPNVTFT-FFNTSKSNSSL
(38.8%) Db_B5GT 1 -----MGTHSTAPDLHVVFFPFLAHGHMIPSLDIAKLF--AARGVKTT-IITTPLNASMF
(21.2%) Cm_FG2RT 1 ------MDTKHQDKPSILMLPWLAHGHIAPHLELAKKL--SQKNFHIY-FCSTPNNLQSF
(20.2%) BpUGT94B1 1 -----MDSKIDSKTFRVVMLPWLAYSHISRFLVFAKRL--TNHNFHIY-ICSSQTNMQYL
(19.2%) Ph_3RT 1 ---MENEMKHSNDALHVVMFPFFAFGHISPFVQLANKL--SSYGVKVS-FFTASGNASRV
(21.3%) Si__UGT71A9 1 -------MSADQKLTSLVFVPFPIMSHLATAVKTAKLLADRDERLSIT-VLVMKLPIDTL
(20.9%) Si_UGT94D1 1 -------MDTRKRSIRILMFPWLAHGHISAFLELAKSL--AKRNFVIY-ICSSQVNLNSI
(22.2%) At_UGT71B6 1 ------------MKIELVFIPSPAISHLMATVEMAEQLVDKNDNLSIT-VIIISFSSKNT
(52.5%) At_UGT73C6 1 ---MAFEKNNEPFPLHFVLFPFMAQGHMIPMVDIARLL--AQRGVLIT-IVTTPHNAARF
(36.8%) At_UGT73B1 1 -----MGTPVEVSKLHFLLFPFMAHGHMIPTLDMAKLF--ATKGAKST-ILTTPLNAKLF
(22.2%) At_UGT75C1 1 ---MATSVNGSHRRPHYLLVTFPAQGHINPALQLANRL--IHHGATVT-YSTAVSAHRRM
(22.4%) At_UGT78D2 1 ----MTKPSDPTRDSHVAVLAFPFGTHAAPLLTVTRRLASASPSTVFS-FFNTAQSNSSL
(34.5%) Gm_UGT73P2 1 -------MEKKKGELKSIFLPFLSTSHIIPLVDMARLF--ALHDVDVT-IITTAHNATVF
(22.3%) Gm_UGT91H4 1 MDSVALNGKSNDKPLHVAMLPWLAMGHIYPYFEVAKIL--AQKGHFVT-FINSPKNIDRM
(22.2%) AcGaT 1 --------MGSSAEPHVGVLAFPFATHAGLLLGLVRRLAAAAPNVNFS-FYSTAASNRSL
(25.5%) MtUGT85h2 1 ------MGNFANRKPHVVMIPYPVQGHINPLFKLAKLL--HLRGFHIT-FVNTEYNHKRL
(26.2%) GhUGT1 1 -------MEKQQKSGHLVLVMAPFQGHLTPMLQLATIL--HSKGFSIT-IVHPELNSLNP
(24.3%) AtUGT72B1 1 --------MEESKTPHVAIIPSPGMGHLIPLVEFAKRLV-HLHGLTVTFVIAGEGPPSKA
(23.6%) VvUF3GT 1 -------MSQTTTNPHVAVLAFPFSTHAAPLLAVVRRLAAAAPHAVFSFFSTSQSNASIF
(21.2%) MtUGT71g1 1 -----MSMSDINKNSELIFIPAPGIGHLASALEFAKLLTNHDKNLYIT-VFCIKFPGMPF
(23.8%) MtUGT78g1 1 -MSTFKNEMNGNNLLHVAVLAFPFGTHAAPLLSLVKKIATEAPKVTFSFFCTTTTNDTLF
(25.5%) PL_ZOG1 1 -MALNDKSIPHETKVVVLLIPFPAQGHLNQFLHLSRLI--VAQNIPVH-YVGTVTHIRQA
(24.6%) MtUGT72L1 1 ---MNLASNFMDKTIHIAVVPGVGYGHLVPILHFSKLLIQLHPDIHVTCIIPTLGSPPSS
(34.5%) MtUGT73F3 1 -----MEGVEVEQPLKVYFIPFLASGHMIPLFDIATMF--ASRGQQVT-VITTPANAKSL
(33.8%) MtUGT73K1 1 -------MGTESKPLKIYMLPFFAQGHLIPLVNLARLV--ASKNQHVT-IITTPSNAQLF
(24.3%) MtUGT88E2 1 ------------MKDTLVLYPALGKGHLNSMIELGKLILTHNPSYSITILILTPPNTTLQ
(23.0%) MtUGT88E1 1 ------------MKDTIVLYPAFGSGHLMSMVELGKLILTHHPSFSIKILILTPPNQDTN
(47.8%) MtUGT73C8 1 -------MVSQDPKVHFVLFPMMAQGHMIPMMDIAKILA-QHQNVIVT-IVTTPKNASRF
(53.2%) AtUGT73C5 1 ----MVSETTKSSPLHFVLFPFMAQGHMIPMVDIARLL--AQRGVIIT-IVTTPHNAARF
(53.3%) AtUGT73C1 1 ------MASEFRPPLHFVLFPFMAQGHMIPMVDIARLL--AQRGVTIT-IVTTPQNAGRF
(22.5%) AtUGT76C1 1 --------MEKRNERQVILFPLPLQGCINPMLQLAKIL--YSRGFSIT-IIHTRFNAPKS
(23.8%) AtUGT76C2 1 -------MEEKRNGLRVILFPLPLQGCINPMLQLANIL--HVRGFSIT-VIHTRFNAPKA
(25.9%) AtUGT85A1 1 ---MGSQIIHNSQKPHVVCVPYPAQGHINPMMRVAKLL--HARGFYVT-FVNTVYNHNRF
(32.8%) Va_AOG 1 -------MKTLTPSVEIFFFPYVGGGHQIPMIDAARMF--ASHGASST-ILATPSTTPLF

GhUGT73C14 47 QKSVARAIESGLP----IRLLQLQFPGKEVGLIDGVENIDML---HSMEDLIKFVS---A
Zm_iaglu 46 D----------------VDAHPAMVEAISDGHDEGGFA-------SAAGVAEYLEK---Q
Gtr_UGT78B1 50 FSPTNLI----------SIGSNIKPYAVWDGSPEGFVFSGN----PREPIEYFLNA---A
Nt_UGT73A1 47 SKAIQRNKHLGIE----IEIRLIKFPAVENGLPEECERLDQI---PSDEKLPNFFK---A
Pv_ZOX1 52 KLRYHNATSN-------IHFHAFEVPPYVSPPPNPEDDF------PSHLIPSFE-----A
Pf_A5GT 47 ANTASAAA---------GNPPGLDFVAFSDGYDDGLK--------PCGDGKRYMSE---M
Sb_HMNGT 54 LRAKGEAAVRPPA----TSSARFRIEVIDDGLSLSVPQND-----VGGLVDSLRKN---C
Ph_F3GalT 49 FTTPHDN--------------NIKPFNISDGVPEGYVVGKGG---IEALIGLFFKS---A
Db_B5GT 53 TKAIEKTRKNTETQ---MEIEVFSFPSEEAGLPLGCENLEQA---MAIGANNEFFN---A
Cm_FG2RT 52 GRNVEKNFSSS------IQLIELQLPNTFPELPSQNQTTKN----LPPHLIYTLVG---A
BpUGT94B1 53 KNNLTSQYSKS------IQLIELNLPSSSE-LPLQYHTTHG----LPPHLTKTLSD---D
Ph_3RT 55 KSMLNSAPT--------THIVPLTLPHV-EGLPPGAESTAE----LTPASAELLKV---A
Si__UGT71A9 53 ISSYTKNSPD-------ARVKVVQLPEDEPTFTKLMKS-------SKNFFFRYIES---Q
Si_UGT94D1 51 SKNMSSKDS--------ISVKLVELHIPTTILPPPYHTTNG----LPPHLMSTLKR---A
At_UGT71B6 48 SMITSL-----------TSNNRLRYEIISGGDQQPTEL--KA---TDSHIQSL-------
At_UGT73C6 55 KNVLNRAIESGLP----INLVQVKFPYQEAGLQEGQENMDLL---TTMEQITSFFK---A
At_UGT73B1 53 FEKPIKSFNQDNPGLEDITIQILNFPCTELGLPDGCENTDFIFSTPDLNVGDLSQKFLLA
At_UGT75C1 55 GEPPSTK--------------GLSFAWFTDGFDDGLKSFED----QKIYMSELKRC---G
At_UGT78D2 56 FSSGDEA----------DRPANIRVYDIADGVPEGYVFSGR----PQEAIELFLQA---A
Gm_UGT73P2 51 QKSIDLDASRGRP----IRTHVVNFPAAQVGLPVGIEAFNVD---TPREMTPRIYM---G
Gm_UGT91H4 58 PKTPKHLEPF-------IKLVKLPLPKI-EHLPEGAESTMD----IPSKKNCFLKK---A
AcGaT 52 FSYPN------------SPYSNVIPYDVSDGVPEGYVFSGK----PQEDINLFLTV---A
MtUGT85h2 52 LKSRGPKAF--------DGFTDFNFESIPDGLTPMEGDGD-----VSQDVPTLCQS---V
GhUGT1 51 SNHPEFTFVPIPDK---IKESQLSDEDLADKLKESLVSTVD----VAGSVQSLNKN---C
AtUGT72B1 52 QRTVLDSLPSSISS---VFLPPVDLTDLSSSTR------------IESRISLTVTR---S
VvUF3GT 54 HDSMH------------TMQCNIKSYDISDGVPEGYVFAGR----PQEDIELFTRA---A
MtUGT71g1 55 ADSYIKSVL--------ASQPQIQLIDLPEVEPPPQELLKS----PEFYILTFLES---L
MtUGT78g1 60 SRSN-------------EFLPNIKYYNVHDGLPKGYVSSGN----PREPIFLFIKA---M
PL_ZOG1 57 TLRYNNPTSN-------IHFHAFQVPPFVSPPPNPEDDF------PSHLIPSF--E---A
MtUGT72L1 58 SETILQTLPSNIDY---MFLPEVQPSDLPQGLP------------MEIQIQLTVTN---S
MtUGT73F3 53 TKSLSSDAPSF------LRLHTVDFPSQQVGLPEGIESMSST---TDPTTTWKIHT---G
MtUGT73K1 51 DKTIEEEKAAGHH----IRVHIIKFPSAQLGLPTGVENLFAA---SDNQTAGKIHM---A
MtUGT88E2 49 PPQEIQKLTTT------TTFGCESFPSITFHHIPPISFPVTL---PPHIVPLEVCG--RS
MtUGT88E1 49 TINVSTSQY--------ISSVSNKFPSINFHYIPSISFTFTL---PPHLQTLELSP--RS
MtUGT73C8 52 TSIVARCVEYGLD----IQLVQLEFPCKESGLPEGCENLDML---PALGMASNFLN---A
AtUGT73C5 54 KNVLNRAIESGLP----INLVQVKFPYLEAGLQEGQENIDSL---DTMERMIPFFK---A
AtUGT73C1 52 KNVLSRAIQSGLP----INLVQVKFPSQESGSPEGQENLDLL---DSLGASLTFFK---A
AtUGT76C1 50 -----------------SDHPLFTFLQIRDGLSESQTQSRD----LLLQLTLLNNN---C
AtUGT76C2 51 -----------------SSHPLFTFLQIPDGLSETEIQDG-----VMSLLAQINLN---A
AtUGT85A1 55 LRSRGSNAL--------DGLPSFRFESIADGLPETDMDATQD---ITALCESTMKN---C
Va_AOG 51 QKCITRDQ---------KFGLPISIHTLSADVPQ-----------SDISVGPFLDT----

GhUGT73C14 97 ANKMEEAMLKLFE-KLT-----PRPNCIISDINLFYTRKIASKFQVPKISFHGFCCFCLL
Zm_iaglu 80 AAAASASLASLVE-ARASSA--DAFTCVVYDSYEDWVLPVARRMGLPAVPFSTQSCAVSA
Gtr_UGT78B1 93 PDNFDKAMKKAVE-DTG-----VNISCLLTDAFLWFAADFSEKIGVPWIPVWTAASCSLC
Nt_UGT73A1 97 VAMMQEPLEQLIE-E-------CRPDCLISDMFLPWTTDTAAKFNIPRIVFHGTSFFALC
Pv_ZOX1 94 SAHLREPVGKLLQ-SLSSQA--KRVVLINDSLMASVAQDAANFSNVERYCFQVFSALNTA
Pf_A5GT 87 KARGSEALRNLLL-NN------HDVTFVVYSHLFAWAAEVARESQVPSALLWVEPATVLC
Sb_HMNGT 102 LHPFRALLRRLGQ-EVEGQDA-PPVTCVVGDVVMTFAAAAAREAGIPEVQFFTASACGLL
Ph_F3GalT 89 KENIQNAMAAAVE-ESG-----KKITCVMADAFMWFSGEIAEELSVGWIPLWTSAAGSLS
Db_B5GT 104 ANLLKEQLENFLV-K-------TRPNCLVADMFFTWAADSTAKFNIPTLVFHGFSFFAQC
Cm_FG2RT 99 FEDAKPAFCNILE-T-------LKPTLVMYDLFQPMAAEAAYQYDIAAILFLPLSAVACS
BpUGT94B1 99 YQKSGPDFETILI-K-------LNPHLVIYDFNQLWAPEVASTLHIPSIQLLSGCVALYA
Ph_3RT 99 LDLMQPQIKTLLS-H-------LKPHFVLFDFAQEWLPKMANGLGIKTVYYSVVVALSTA
Si__UGT71A9 96 KGTVRDAVAEIMK-SSRS----CRLAGFVIDMFCTTMIDVANELGVPTYMFFSSGSATLG
Si_UGT94D1 96 LDSARPAFSTLLQ-T-------LKPDLVLYDFLQSWASEEAESQNIPAMVFLSTGAAAIS
At_UGT71B6 85 KPLVRDAVAKLVD-STLPDA--PRLAGFVVDMYCTSMIDVANEFGVPSYLFYTSNAGFLG
At_UGT73C6 105 VNLLKEPVQNLIE-EMS-----PRPSCLISDMCLSYTSEIAKKFKIPKILFHGMGCFCLL
At_UGT73B1 113 MKYFEEPLEELLV-T-------MRPDCLVGNMFFPWSTKVAEKFGVPRLVFHGTGYFSLC
At_UGT75C1 94 SNALRDIIKANLD-ATTET---EPITGVIYSVLVPWVSTVAREFHLPTTLLWIEPATVLD
At_UGT78D2 99 PENFRREIAKAET-EVG-----TEVKCLMTDAFFWFAADMATEINASWIAFWTAGANSLS
Gm_UGT73P2 101 LSLLQQVFEKLFH-D-------LQPDFIVTDMFHPWSVDAAAKLGIPRIMFHGASYLARS
Gm_UGT91H4 103 YEGLQYAVSKLLK-T-------SNPDWVLYDFAAAWVIPIAKSYNIPCAHYNITPAFNKV
AcGaT 93 SDEFKRGLEKAAV-DSG-----RKITCLVADAFLWFSGDLAEQIRVPWVPLWTSGACSLS
MtUGT85h2 96 RKNFLKPYCELLT-RLNHSTNVPPVTCLVSDCCMSFTIQAAEEFELPNVLYFSSSACSLL
GhUGT1 101 AAPLKKCLENILH-SH------HHIAAVIYDTLMFCAQTIVNDLGLPGITLRTSSATTLL
AtUGT72B1 94 NPELRKVFDSFVE-GG------RLPTALVVDLFGTDAFDVAVEFHVPPYIFYPTTANVLS
VvUF3GT 95 PESFRQGMVMAVA-ETG-----RPVSCLVADAFIWFAADMAAEMGVAWLPFWTAGPNSLS
MtUGT71g1 100 IPHVKATIKTILS---------NKVVGLVLDFFCVSMIDVGNEFGIPSYLFLTSNVGFLS
MtUGT78g1 100 QENFKHVIDEAVA-ETG-----KNITCLVTDAFFWFGADLAEEMHAKWVPLWTAGPHSLL
PL_ZOG1 99 SAHLREPVGKLLQ-SLSSQA--KRVVVINDSLMASVAQDAANISNVENYTFHSFSAFNTS
MtUGT72L1 100 LPYLHEALKSLAL-R-------IPLVALVVDAFAVEALNFAKEFNMLSYIYFCAAASTLA
MtUGT73F3 101 AMLLKEPIGDFIE-N-------DPPDCIISDSTYPWVNDLADKFQIPNITFNGLCLFAVS
MtUGT73K1 101 AHFVKADIEEFMK-E-------NPPDVFISDIIFTWSESTAKNLQIPRLVFNPISIFDVC
MtUGT88E2 98 NHHVNHVLQSISK-T-------SNLKGVILDFMNYSTNQITSTLDIPTYFFYTSGASTLA
MtUGT88E1 96 NHHVHHILQSIAK-T-------SNLKAVMLDFLNYSASQVTNNLEIPTYFYYTSGASLLC
MtUGT73C8 102 LKFFQQEVEKLFE-EFT-----TPATCIISDMCLPYTSHVARKFNIPRITFLGVSCFHLF
AtUGT73C5 104 VNFLEEPVQKLIE-EMN-----PRPSCLISDFCLPYTSKIAKKFNIPKILFHGMGCFCLL
AtUGT73C1 102 FSLLEEPVEKLLK-EIQ-----PRPNCIIADMCLPYTNRIAKNLGIPKIIFHGMCCFNLL
AtUGT76C1 86 QIPFRECLAKLIKPSSDSGTEDRKISCVIDDSGWVFTQSVAESFNLPRFVLCAYKFSFFL
AtUGT76C2 86 ESPFRDCLRKVLL-ESKES---ERVTCLIDDCGWLFTQSVSESLKLPRLVLCTFKATFFN
AtUGT85A1 101 LAPFRELLQRINA-GDNV----PPVSCIVSDGCMSFTLDVAEELGVPEVLFWTTSGCAFL
Va_AOG 87 -SALLEPLRQLLL-Q-------RRPHCIVVDMFHRWSGDVVYELGIPRTLFNGIGCFALC

GhUGT73C14 151 CLRNIQSSKINETVTSD------------SEYFTVPG-LTDKVEFTRVQL----PLDNDG
Zm_iaglu 137 VYYHFSQGRLA-VPPGAAADGSDGGAGAAALSEAFLG-LP-EMERSELPS----FVFDHG
Gtr_UGT78B1 147 LHVYTDEIRSRFAEFDIAEKAE-------KTIDFIPG-LS-AISFSDLPE-ELIMEDSQS
Nt_UGT73A1 149 VENSVRLNKPFKNVSSD------------SETFVVPD-LPHEIKLTRTQVSPFERSGEET
Pv_ZOX1 151 GDFWEQMGKPP------------------LADFHFPD-IP------------SLQGCISA
Pf_A5GT 140 IYYFYFNGYADEIDAG-------------SDEIQLPR-LP-PLEQRSLPT--FLLPETPE
Sb_HMNGT 160 GYLHYGELVERGLVPFRDASLLADDDYLDTPLEWVPG-MS-HMRLRDMPT-FCRTTDPDD
Ph_F3GalT 143 VHVYTDLIRENVEAQGIAGRED-------EILTFIPG-FA-ELRLGSLPS-GVVSGDLES
Db_B5GT 156 AKEVMWRYKPYKAVSSD------------TEVFSLPF-LPHEVKMTRLQVPESMRKGEET
Cm_FG2RT 151 FLLHNIV----------------------NPSLKYPF-FESDYQDRESKNINYFLHLTAN
BpUGT94B1 151 LDAHLYTKPLDEN----------------LAKFPFPEIYP---KNRDIPK----------
Ph_3RT 151 FLTCPARVLEPKKYPSLEDMK--------KPPLGFPQ-TS-VTSVRTFEARDFLYVFKSF
Si__UGT71A9 151 LMFHLQSLRDDNNVDVMEYKNS-------DAAISIPT-YVNPVPVAVWPS--PVFEEDSG
Si_UGT94D1 148 FIMYHWFE---------------------TRPEEYP--FP-AIYFREHEYDNFCRFKSSD
At_UGT71B6 142 LLLHIQF--MYDAEDIYDMSELEDS----DVELVVPS-LTSPYPLKCLPY-----IFKSK
At_UGT73C6 159 CVNVLRKNREILDNLKSD-----------KEYFIVPY-FPDRVEFTRPQV-PVETYVPAG
At_UGT73B1 165 ASHCIRL--PKNVATS-------------SEPFVIPD-LPGDILITEEQV---METEEES
At_UGT75C1 150 IYYYYFNTSYKHLFD--------------VEPIKLPK-LP-LITTGDLPS--FLQPSKAL
At_UGT78D2 153 AHLYTDLIRETIGVKEVGERME-------ETIGVISG-ME-KIRVKDTPE-GVVFGNLDS
Gm_UGT73P2 153 AAHSVEQYAPHLEAKFD------------TDKFVLPG-LPDNLEMTRLQLPDWLR--SPN
Gm_UGT91H4 155 FFDPPKDKMKDYSLASIC-----------GPPTWLP--FTTTIHIRPYEFLRAYEGTKDE
AcGaT 147 IHVYTDLIRQTVGLGGIEGRMD-------EILTFIPG-FS-ELRLGDLPG-GVLFGNLES
MtUGT85h2 155 NVMHFRSFVERGIIPFKDESYLTNGCLE-TKVDWIPG-LK-NFRLKDIVD-FIRTTNPND
GhUGT1 154 LFPVLPQLGEKEL----------------MSGIESPE-LQ-ALQLQRLRA--LIVQNPTQ
AtUGT72B1 147 FFLHLPKLDETVSCEFREL----------TEPLMLPGCVP--VAGKDFLDPAQDRKDDAY
VvUF3GT 149 THVYIDEIREKIGVSGIQGRED-------ELLNFIPG-MS-KVRFRDLQE-GIVFGNLNS
MtUGT71g1 151 LMLSLKNRQIEEVFDDSDRD---------HQLLNIPG-ISNQVPSNVLPD---ACFNKDG
MtUGT78g1 154 THVYTDLIREKTGSKEVHDV---------KSIDVLPG-FP-ELKASDLPE--GVIKDIDV
PL_ZOG1 156 GDFWEEMGKPP------------------VGDFHFPE-FP------------SLEGCIAA
MtUGT72L1 152 WSFYLPKLDEETTCEYRDL----------PEPIKVPG-CV-PLHGRDLLT---IVQDRSS
MtUGT73F3 153 LVETLKTNNLLKSQTDSDSD---------SSSFVVPN-FPHHITLCGKPP---------K
MtUGT73K1 153 MIQAIQS--HPESFVSD------------SGPYQIHG-LPHPLTLPIKPS---------P
MtUGT88E2 150 VFLQLPTIHQSTTKSLKEF----------HMYPRIPG-LP-LVPIVDMPD---EVKDRES
MtUGT88E1 148 LFLNFPTFHKNATIPIKDYNM--------HTPIELPG-LP-RLSKEDYPD---EGKDPSS
MtUGT73C8 156 NMHNFHVNNMAEIMANKE-----------SEYFELPG-IPDKIEMTIAQT--GLGGLKGE
AtUGT73C5 158 CMHVLRKNREILDNLKSD-----------KELFTVPD-FPDRVEFTRTQV-PVETYVPAG
AtUGT73C1 156 CTHIMHQNHEFLETIESD-----------KEYFPIPN-FPDRVEFTKSQL-PM--VLVAG
AtUGT76C1 146 GHFLVPQIRREGFLPVPDS----------EADDLVPE-FP-PLRKKDLSR-IMGTSAQSK
AtUGT76C2 142 AYPSLPLIRTKGYLPVSES----------EAEDSVPE-FP-PLQKRDLSKVFGEFGEKLD
AtUGT85A1 156 AYLHFYLFIEKGLCPLKDESYLTKEYLEDTVIDFIPT-MK-NVKLKDIPS-FIRTTNPDD
Va_AOG 138 VQENLRHVAFKSVSTD-------------SEPFLVPN-IPDRIEMTMSQLPPFLRNPSGI

GhUGT73C14 194 SWKEI-FEPMWEADRASYG--VVINTFEELE-SAYVKEYRK-EKKA--------WCIG--
Zm_iaglu 190 PYPTIAMQAIKQFAHAGKDDWVLFNSFEELE-TEVLAGLTK-YLKA--------RAIG--
Gtr_UGT78B1 197 IFALT-LHNMGLKLHKATA--VAVNSFEEID-PIITNHLRS-TNQLN-I-----LNIG--
Nt_UGT73A1 196 AMTRM-IKTVRESDSKSYG--VVFNSFYELE-TDYVEHYTK-VLGRR-A-----WAIG--
Pv_ZOX1 180 QFTDF-LTAQNEFRKFNNG--DIYNTSRVIE-GPYVELLER-FNGGKEV-----WALG--
Pf_A5GT 183 RFRLM-MKEKLETLDGEEKAKVLVNTFDALE-PDALTAIDRYEL----------IGIG--
Sb_HMNGT 217 VMVSA-TLQQMESAAGSKA--LILNTLYELE-KDVVDALAA-FFPPI-------YTVG--
Ph_F3GalT 193 PFSVM-LHKMGKTIGKATA--LPVNSFEELD-PPIVEDLKS-KFNNF-------LNVG--
Db_B5GT 203 HFTKR-TERIRELERKSYG--VIVNSFYELE-PDYADFLRK-ELGRR-A-----WHIG--
Cm_FG2RT 188 GTLN--KDRFLKAFELSCK-FVFIKTSREIE-SKYLDYFPS-LMGNE-I-----IPVG--
BpUGT94B1 182 GGSKY-IERFVDCMRRSCE-IILVRSTMELE-GKYIDYLSK-TLGKK-V-----LPVG--
Ph_3RT 201 HNGPTLYDRIQSGLRGCSA--ILAKTCSQME-GPYIKYVEA-QFNKP-V-----FSNR--
Si__UGT71A9 201 ------FLDFAKRFRETKG--IIVNTFLEFE-THQIRSLSD-DKKIPPV-----YPVG--
Si_UGT94D1 184 SGTSD-QLRVSDCVKRSHD-LVLIKTFRELE-GQYVDFLSD-LTRKR-F-----VPVG--
At_UGT71B6 190 EWLTF-FVTQARRFRETKG--ILVNTVPDLE-PQALTFLSN-GNIPR-A-----YPVG--
At_UGT73C6 206 -WKEI-LEDMVEADKTSYG--VIVNSFQELE-PAYAKDFKEARSGKA-------WTIG--
At_UGT73B1 206 VMGRF-MKAIRDSERDSFG--VLVNSFYELE-QAYSDYFKS-FVAKR-A-----WHIG--
At_UGT75C1 192 PSALVTLREHIEALETESNPKILVNTFSALE-HDALTSVEK----LK-M-----IPIG--
At_UGT78D2 203 VFSKM-LHQMGLALPRATA--VFINSFEDLD-PTLTNNLRS-RFKRY-------LNIG--
Gm_UGT73P2 198 QYTEL-MRTIKQSEKKSYG--SLFNSFYDLE-SAYYEHYKS-IMGTK-S-----WGIG--
Gm_UGT91H4 202 ETGERASFDLNKAYSSCDL--FLLRTSRELE-GDWLDYLAG-NYKVP-V-----VPVGLL
AcGaT 197 PFSIM-LHKMGQTLPRAAA--VPINSFEELD-PDLMKDIKS-KFKKI-------LNVG--
MtUGT85h2 211 IMLEF-FIEVADRVNKDTT--ILLNTFNELE-SDVINALSS---TIPSI-----YPIG--
GhUGT1 194 AMMEV-RAAFTNAMKFSSA--IIVNSMEFLE-LEALSKVRQ-YFRTP-I-----FIVG--
AtUGT72B1 195 KW----LLHNTKRYKEAEG--ILVNTFFELE-PNAIKALQEPGLDKPPV-----YPVG--
VvUF3GT 199 LFSRM-LHRMGQVLPKATA--VFINSFEELD-DSLTNDLKS-KLKTY-------LNIG--
MtUGT71g1 198 GYIA--YYKLAERFRDTKG--IIVNTFSDLE-QSSIDALYDHDEKIPPI-----YAVG--
MtUGT78g1 201 PFATM-LHKMGLELPRANA--VAINSFATIH-PLIENELNS-KFKLL-------LNVG--
PL_ZOG1 185 QFKGF-RTAQYEFRKFNNG--DIYNTSRVIE-GPYVELLEL-FNGGKKV-----WALG--
MtUGT72L1 197 QAYKY-FLQHVKSLSFADG--VLVNSFLEMEMGPINALTEE-GSGNPSV-----YPVG--
MtUGT73F3 194 VIGIF-MGMMLETVLKSKA--LIINNFSELDGEECIQHYEK-ATGHK-V-----WHLG--
MtUGT73K1 189 GFARL-TESLIEAENDSHG--VIVNSFAELD-EGYTEYYEN-LTGRK-V-----WHVG--
MtUGT88E2 195 KSYKV-FLDMATSMRESDG--VIINTFDAIE-GRAAKALKA-GLCLPEGTTPPLFCIG--
MtUGT88E1 195 PSYQV-LLQSAKSLRESDG--IIVNTFDAIE-KKAIKALRN-GLCVPDGTTPLLFCIG--
MtUGT73C8 202 VWKQF-NDDLLEAEIGSYG--MLVNSFEELE-PTYARDYKK-VRNDK-V-----WCIG--
AtUGT73C5 205 DWKDI-FDGMVEANETSYG--VIVNSFQELE-PAYAKDYKEVRSGKA-------WTIG--
AtUGT73C1 201 DWKDF-LDGMTEGDNTSYG--VIVNTFEELE-PAYVRDYKKVKAGKI-------WSIG--
AtUGT76C1 193 PLDAY-LLKILDATKPASG--IIVMSCKELD-HDSLAESNK-VFSIP-I-----FPIG--
AtUGT76C2 190 PF----LHAVVETTIRSSG--LIYMSCEELE-KDSLTLSNE-IFKVP-V-----FAIG--
AtUGT85A1 213 VMISF-ALRETERAKRASA--IILNTFDDLE-HDVVHAMQS---ILPPV-----YSVG--
Va_AOG 184 PER---WRGMKQLEEKSFG--TLINSFYDLE-PAYADLIKS-KWGNK-A-----WIVG--

GhUGT73C14 239 -PVSL--SHKDELDMA--ERG---NKTSIDGQK-CLKWLDSQQPGSVIYACLGSIG-TIK
Zm_iaglu 238 -PCVP--LPTAGRTAGANGRITYGANLVKPEDA-CTKWLDTKPDRSVAYVSFGSLA-SLG
Gtr_UGT78B1 244 -PLQT--LSSSIP---------------PEDNE-CLKWLQTQKESSVVYLSFGTVI-NPP
Nt_UGT73A1 243 -PLSM--CNRDIEDKA--ERG---KKSSIDKHE-CLKWLDSKKPSSVVYICFGSVA-NFT
Pv_ZOX1 228 -PFTP--LAVEKKDSI------------GFSHP-CMEWLDKQEPSSVIYVSFGTTT-ALR
Pf_A5GT 229 -PLIPS-AFLDGGDPS--ETSYGGDLFEKSEENNCVEWLDTKPKSSVVYVSFGSVL-RFP
Sb_HMNGT 263 -PLAEV-IASSDSASA--GLAAMDISIWQEDTR-CLSWLDGKPAGSVVYVNFGSMA-VMT
Ph_F3GalT 239 -PFNL--TTPPPSANI------------TDEYG-CIAWLDKQEPGSVAYIGFGTVA-TPP
Db_B5GT 250 -PVSL--CNRSIEDKA--QRG---RQTSIDEDE-CLKWLNSKKPDSVIYICFGSTG-HLI
Cm_FG2RT 235 -PLIQ------EPTFK------------VDDTK-IMDWLSQKEPRSVVYASFGSEY-FPS
BpUGT94B1 230 -PLV------QEASLL------------QDDHIWIMKWLDKKEESSVVFVCFGSEY-ILS
Ph_3RT 249 -------TRSSGPASG------------KLEEK-WATWLNKFEGGTVIYCSFGSET-FLT
Si__UGT71A9 244 -PILQ--ADENKIEQE------------KEKHAEIMRWLDKQPDSSVVFLCFGTHG-CLE
Si_UGT94D1 232 -PL----VQEVGCDME------------NEGND-IIEWLDGKDRRSTVFSSFGSEY-FLS
At_UGT71B6 237 -PLLH--LKNVNCDYV-----------DKKQSE-ILRWLDEQPPRSVVFLCFGSMG-GFS
At_UGT73C6 252 -PVSL--CNKVGVDKA--ERG---NKSDIDQDE-CLEWLDSKEPGSVLYVCLGSIC-NLP
At_UGT73B1 253 -PLSL--GNRKFEEKA--ERG---KKASIDEHE-CLKWLDSKKCDSVIYMAFGTMS-SFK
At_UGT75C1 239 -PLV---SSSEGKTDL----------FKSSDED-YTKWLDSKLERSVIYISLGTHADDLP
At_UGT78D2 249 -PLGL--LSSTLQQLV------------QDPHG-CLAWMEKRSSGSVAYISFGTVM-TPP
Gm_UGT73P2 245 -PVSLW-ANQDAQDKA--ARG--YAKEEEEKEG-WLKWLNSKAESSVLYVSFGSIN-KFP
Gm_UGT91H4 252 PPSMQI-RDVEEEDNN------------PDWVR-IKDWLDTQESSSVVYIGFGSEL-KLS
AcGaT 243 -PFNL--TSPPPSSNS-------------DEHG-CIPWLDNQNPKSVAYIAFGTVA-TPP
MtUGT85h2 257 -PLPS--LLKQTPQIH--QLDSLDSNLWKEDTE-CLDWLESKEPGSVVYVNFGSTT-VMT
GhUGT1 241 -PLHKL-APAICGSLL------------TEDDK-CISWLNKQAPKSVIYVSLGSIA-NID
AtUGT72B1 241 -PLVN--IGKQEAKQT-------------EESE-CLKWLDNQPLGSVLYVSFGSGG-TLT
VvUF3GT 245 -PFNL--ITP--PPVV------------PNTTG-CLQWLKERKPTSVVYISFGTVT-TPP
MtUGT71g1 246 -PLLD--LKGQPNPKL-----------DQAQHDLILKWLDEQPDKSVVFLCFGSMGVSFG
MtUGT78g1 247 -PFNL----TTPQRKV------------SDEHG-CLEWLDQHENSSVVYISFGSVV-TPP
PL_ZOG1 233 -PFNP--LAVEKKDSI------------GFRHP-CMEWLDKQEPSSVIYISFGTTT-ALR
MtUGT72L1 246 -PIIQ--TVTGSVDDA-------------NGLE-CLSWLDKQQSCSVLYVSFGSGG-TLS
MtUGT73F3 242 -PTSL--IRKTAQEKS--ERG---NEGAVNVHE-SLSWLDSERVNSVLYICFGSIN-YFS
MtUGT73K1 236 -PTSL--MVEIPKKKKVVSTE---NDSSITKHQ-SLTWLDTKEPSSVLYISFGSLC-RLS
MtUGT88E2 248 -PMIS--PPCKGEDER--------------GSS-CLSWLDSQPSQSVVLLSFGSMG-RFS
MtUGT88E1 248 -PVVS--TSCEEDKSG------------------CLSWLDSQPGQSVVLLSFGSLG-RFS
MtUGT73C8 249 -PVSL--SNTDYLDKV--QRGNNNNKVSNDEWE-HLKWLDSHKQGSVIYACFGSLC-NLT
AtUGT73C5 252 -PVSL--CNKVGADKA--ERG---NKSDIDQDE-CLKWLDSKKHGSVLYVCLGSIC-NLP
AtUGT73C1 248 -PVSL--CNKLGEDQA--ERG---NKADIDQDE-CIKWLDSKEEGSVLYVCLGSIC-NLP
AtUGT76C1 240 -PFHIHDVPASSSSLL------------EPDQS-CIPWLDMRETRSVVYVSLGSIA-SLN
AtUGT76C2 234 -PFHSY-FSASSSSLF------------TQDET-CILWLDDQEDKSVIYVSLGSVV-NIT
AtUGT85A1 259 -PLHLL-ANREIEEGS--EIGMMSSNLWKEEME-CLDWLDTKTQNSVIYINFGSIT-VLS
Va_AOG 229 -PVSF--CNRSKEDKT--ERG---KPPTIDEQN-CLNWLNSKKPSSVLYASFGSLA-RLP

GhUGT73C14 289 CPELIELGLGLEASNKPFIWVLRGN----------NPTASEVYK-----WIRRNEFEERT
Zm_iaglu 293 NAQKEELARGLLAAGKPFLWVVRAS----------------DEH-----QVPR-YLLAEA
Gtr_UGT78B1 284 PNEMAALASTLESRKIPFLWSLRDE----------------ARK-----HLPE-NFIDRT
Nt_UGT73A1 293 ASQLHELAMGVEASGQEFIWVVRTE--------------LDNED-----WLPE-GFEERT
Pv_ZOX1 271 DEQIQELATGLEQSKQKFIWVLRDA------DKGDIFDGSEAKRY----ELPE-GFEERV
Pf_A5GT 284 KAQMEEIGKGLLACGRPFLWMIREQ---------KNDDGEEEEE-----ELSCIGELKKM
Sb_HMNGT 317 AAQAREFALGLASCGSPFLWVKRPD------------VVEGEEV-----LLPE-ALLDEV
Ph_F3GalT 282 PNELKAMAEALEESKTPFLWSLKDL----------------FKS-----FFPE-GFLERT
Db_B5GT 300 APQLHEIATALEASGQDFIWAVRGD-----------HGQGNSEE-----WLPP-GYEHRL
Cm_FG2RT 274 TDEIHDIAIGLLLTEVNFIWAFRLH----------PDEKMTIEE-----ALPQ-GFAEEI
BpUGT94B1 270 DNEIEDIAYGLELSQVSFVWAIR------------------AKT-----SALN-GFIDRV
Ph_3RT 288 DDQVKELALGLEQTGLPFFLVLNFP--------ANVDVSAELNR-----ALPE-GFLERV
Si__UGT71A9 288 GDQVKEIAVALENSGHRFLWSLRKP--PPKEKVEFPGEYENSEE-----VLPE-GFLGRT
Si_UGT94D1 273 ANEIEEIAYGLELSGLNFIWVVRFP---------HGDEKIKIEE-----KLPE-GFLERV
At_UGT71B6 281 EEQVRETALALDRSGHRFLWSLRRA--SPNILREPPGEFTNLEE-----ILPE-GFFDRT
At_UGT73C6 302 LSQLLELGLGLEESQRPFIWVIRGW-----------EKYKELVE-----WFSESGFEDRI
At_UGT73B1 303 NEQLIEIAAGLDMSGHDFVWVVNRK-----------GSQVEKED-----WLPE-GFEEKT
At_UGT75C1 284 EKHMEALTHGVLATNRPFLWIVREK---------------NPEE-----KKKN-RFLELI
At_UGT78D2 292 PGELAAIAEGLESSKVPFVWSLKEK----------------SLV-----QLPK-GFLDRT
Gm_UGT73P2 297 YSQLVEIARALEDSGHDFIWVVRKN------------DGGEGDN-----FLEE--FEKRM
Gm_UGT91H4 297 QEDLTELAHGIELSNLPFFWALKNL--------------KEGVL-----ELPE-GFEERT
AcGaT 285 PNELVSLAEALEESGTPFLWSLKDN----------------FKN-----HLPK-GFLERN
MtUGT85h2 310 PEQLLEFAWGLANCKKSFLWIIRPD------------LVIGGSV-----IFSS-EFTNEI
GhUGT1 285 KQELIETAWGLSNSKQPFLWVVRPG----------MVCGSEWIE-----SLSN-GFEENV
AtUGT72B1 283 CEQLNELALGLADSEQRFLWVIRSPSGIANSSYFDSHSQTDPLT-----FLPP-GFLERT
VvUF3GT 286 PAEVVALSEALEASRVPFIWSLRDK----------------ARV-----HLPE-GFLEKT
MtUGT71g1 292 PSQIREIALGLKHSGVRFLWSNSAE-----------------KK-----VFPE-GFLEWM
MtUGT78g1 288 PHELTALAESLEECGFPFIWSFRGD----------------PKE-----KLPK-GFLERT
PL_ZOG1 276 DEQIQQIATGLEQSKQKFIWVLREA------DKGDIFAGSEAKRY----ELPK-GFEERV
MtUGT72L1 288 HEQIVELALGLELSNQKFLWVVRAP----SSSSSNAAYLSAQNDVDALQFLPS-GFLERT
MtUGT73F3 292 DKQLYEMACAIEASGHPFIWVVPEK------KGKEDESEEEKEK-----WLPK-GFEERN
MtUGT73K1 288 NEQLKEMANGIEASKHQFLWVVHGK------------EGEDEDN-----WLPK-GFVERM
MtUGT88E2 289 RAQLNEIAIGLEKSEQRFLWVVRSE---------PDSDKLSLDE-----LFPE-GFLERT
MtUGT88E1 286 KAQINQIAIGLEKSEQRFLWIVRSD---------MESEELSLDE-----LLPE-GFLERT
MtUGT73C8 302 PPQLIELGLALEATKRPFIWVLREG-----------NQLEELKK-----WLEESGFEGRI
AtUGT73C5 302 LSQLKELGLGLEESQRPFIWVIRGW-----------EKYKELVE-----WFSESGFEDRI
AtUGT73C1 298 LSQLKELGLGLEESQRPFIWVIRGW-----------EKYNELLE-----WISESGYKERI
AtUGT76C1 285 ESDFLEIACGLRNTNQSFLWVVRPG----------SVHGRDWIE-----SLPS-GFMESL
AtUGT76C2 278 ETEFLEIACGLSNSKQPFLWVVRPG----------SVLGAKWIE-----PLSE-GLVSSL
AtUGT85A1 313 VKQLVEFAWGLAGSGKEFLWVIRPD------------LVAGEEA-----MVPP-DFLMET
Va_AOG 279 PEQLKEIAYGLEASEQSFIWVVGNI----------LHNPSENKENGSGNWLPE-GFEQRM

**P S P G**

GhUGT73C14 334 K--GR-GLVVVGWAPQVLILSHPAIGGFLTHCGWNSIIEGISAGVPLITFPFMGDQFCNE
Zm_iaglu 331 TATGA-AMVVP-WCPQLDVLAHPAVGCFVTHCGWNSTLEALSFGVPMVAMALWTDQPTNA
Gtr_UGT78B1 322 S--TF-GKIVS-WAPQLHVLENPAIGVFVTHCGWNSTLESIFCRVPVIGRPFFGDQKVNA
Nt_UGT73A1 333 K--EK-GLIIRGWAPQVLILDHESVGAFVTHCGWNSTLEGVSGGVPMVTWPVFAEQFFNE
Pv_ZOX1 320 E--GM-GLVVRDWAPQMEILSHSSTGGFMSHCGWNSCLESLTRGVPMATWAMHSDQPRNA
Pf_A5GT 330 ------GKIVS-WCSQLEVLAHPALGCFVTHCGWNSAVESLSCGVPVVAVPQWFDQTTNA
Sb_HMNGT 359 AR-GR-GLVVP-WCPQAAVLKHAAVGLFVSHCGWNSLLEATAAGQPVLAWPCHGEQTTNC
Ph_F3GalT 320 S--EY-GKIVS-WAPQVQVLSHGSVGVFINHCGWNSVLESIAAGVPVICRPFFGDHQLNA
Db_B5GT 343 Q--GK-GLIIRGWAPQVLILEHEATGGFLTHCGWNSALEGISAGVPMVTWPTFAEQFHNE
Cm_FG2RT 318 ERNNK-GMIVQGWVPQAKILRHGSIGGFLSHCGWGSVVEGMVFGVPIIGVPMAYEQPSNA
BpUGT94B1 306 G--DK-GLVIDKWVPQANILSHSSTGGFISHCGWSSTMESIRYGVPIIAMPMQFDQPYNA
Ph_3RT 334 K--DK-GIIHSGWVQQQNILAHSSVGCYVCHAGFSSVIEALVNDCQVVMLPQKGDQILNA
Si__UGT71A9 340 T--DM-GKVIG-WAPQMAVLSHPAVGGFVSHCGWNSVLESVWCGVPMAVWPLSAEQQANA
Si_UGT94D1 318 E--GR-GLVVEGWAQQRRILSHPSVGGFLSHCGWSSVMEGVYSGVPIIAVPMHLDQPFNA
At_UGT71B6 333 A--NR-GKVIG-WAEQVAILAKPAIGGFVSHGGWNSTLESLWFGVPMAIWPLYAEQKFNA
At_UGT73C6 346 Q--DR-GLLIKGWSPQMLILSHPSVGGFLTHCGWNSTLEGITAGLPMLTWPLFADQFCNE
At_UGT73B1 346 K--GK-GLIIRGWAPQVLILEHKAIGGFLTHCGWNSLLEGVAAGLPMVTWPVGAEQFYNE
At_UGT75C1 323 RGSDR-GLVVG-WCSQTAVLAHCAVGCFVTHCGWNSTLESLESGVPVVAFPQFADQCTTA
At_UGT78D2 330 R--EQ-GIVVP-WAPQVELLKHEATGVFVTHCGWNSVLESVSGGVPMICRPFFGDQRLNG
Gm_UGT73P2 338 KESNK-GYLIWGWAPQLLILENPAIGGLVTHCGWNTVVESVNAGLPMATWPLFAEHFFNE
Gm_UGT91H4 337 K--ER-GIVWKTWAPQLKILAHGAIGGCMSHCGSGSVIEKVHFGHVLVTLPYLLDQCLFS
AcGaT 323 S--KS-GKIVA-WAPQIQVLSHDAVGVVITHGGWNSVVESIAAGVPVICRPFFGDHHINT
MtUGT85h2 352 A--DR-GLIAS-WCPQDKVLNHPSIGGFLTHCGWNSTTESICAGVPMLCWPFFADQPTDC
GhUGT1 329 G--ER-GCIVK-WAPQKEVLAHGAVGGFWSHCGWNSTIESICEGVPMLCRPFFGDQLLNT
AtUGT72B1 337 K--KR-GFVIPFWAPQAQVLAHPSTGGFLTHCGWNSTLESVVSGIPLIAWPLYAEQKMNA
VvUF3GT 324 R--GY-GMVVP-WAPQAEVLAHEAVGAFVTHCGWNSLWESVAGGVPLICRPFFGDQRLNG
MtUGT71g1 329 ELEGK-GMICG-WAPQVEVLAHKAIGGFVSHCGWNSILESMWFGVPILTWPIYAEQQLNA
MtUGT78g1 326 K--TK-GKIVA-WAPQVEILKHSSVGVFLTHSGWNSVLECIVGGVPMISRPFFGDQGLNT
PL_ZOG1 325 E--GM-GLVVRDWAPQLEILSHSSTGGFMSHCGWNSCLESITMGVPIATWPMHSDQPRNA
MtUGT72L1 343 K--EE-GFVITSWAPQIQILSHSSVGGFLSHCGWSSTLESVVHGVPLITWPMFAEQGMNA
MtUGT73F3 340 I--GKKGLIIRGWAPQVKILSHPAVGGFMTHCGGNSTVEAVSAGVPMITWPVHGDQFYNE
MtUGT73K1 330 KEEKK-GMLIKGWVPQALILDHPSIGGFLTHCGWNATVEAISSGVPMVTMPGFGDQYYNE
MtUGT88E2 334 K--DK-GMVVRNWAPQVAILSHNSVGGFVTHCGWNSVLEAICEGVPMIAWPLFAEQRLNR
MtUGT88E1 331 K--EK-GMVVRNWAPQGSILRHSSVGGFVTHCGWNSVLEAICEGVPMITWPLYAEQKMNR
MtUGT73C8 346 N--GR-GLVIKGWAPQLLILSHLAIGGFLTHCGWNSTLEAICAGVPMVTWPLFADQFLNE
AtUGT73C5 346 Q--DR-GLLIKGWSPQMLILSHPSVGGFLTHCGWNSTLEGITAGLPLLTWPLFADQFCNE
AtUGT73C1 342 K--ER-GLLITGWSPQMLILTHPAVGGFLTHCGWNSTLEGITSGVPLLTWPLFGDQFCNE
AtUGT76C1 329 D--GK-GKIVR-WAPQLDVLAHRATGGFLTHNGWNSTLESICEGVPMICLPCKWDQFVNA
AtUGT76C2 322 E--EK-GKIVK-WAPQQEVLAHRATGGFLTHNGWNSTLESICEGVPMICLPGGWDQMLNS
AtUGT85A1 355 K--DR-SMLAS-WCPQEKVLSHPAIGGFLTHCGWNSILESLSCGVPMVCWPFFADQQMNC
Va_AOG 328 KETGK-GLVLRGWAPQLLILEHAAIKGFMTHCGWNSTLEGVSAGVPMITWPLTAEQFSNE

GhUGT73C14 391 KLAVQILKIGVNLGANKPTMLG-DEKSGF---ILNTEHVKNAIDKLME--QGNEGKEMRK
Zm_iaglu 389 RNVELAWGAGVRAR---------RDAGAG---VFLRGEVERCVRAVMD--GGEAASAARK
Gtr_UGT78B1 378 RMVEDVWKIGVGVK-------------GG---VFTEDETTRVLELVLF---SDKGKEMRQ
Nt_UGT73A1 390 KLVTEVLKTGAGVG---SIQW--KRSASE---GVKREAIAKAIKRVMV---SEEADGFRN
Pv_ZOX1 377 VLVTDVLKVGLIVK-----DW---EQRKS---LVSASVIENAVRRLM---ETKEGDEIRK
Pf_A5GT 383 KLIEDAWGTGVRVR----------MNEGG---GVDGSEIERCVEMVMD--GGEKSKLVRE
Sb_HMNGT 416 RQLCEVWGNGAQLP-------------R----EVESGAVARLVREMMV---GDLGKEKRA
Ph_F3GalT 376 WMVEKVWKIGVKIE-------------GG---VFTKDGTMLALDLVLS--KDKRNTELKQ
Db_B5GT 400 QLLTQILKVGVAVG---SKKWTLKPSIED---VIKAEDIEKAVREVMV---GEEGEERRR
Cm_FG2RT 377 KVVVD-NGMGMVVP---------RDKINQ---RLGGEEVARVIKHVVL---QEEAKQIRR
BpUGT94B1 363 RLMET-VGAGIEVG----------RDGEG---RLKREEIAAVVRKVVV---EDSGESIRE
Ph_3RT 391 KLVSGDMEAGVEIN---------RRDEDG---YFGKEDIKEAVEKVMVDVEKDPGKLIRE
Si__UGT71A9 396 FLLVKEFEMAVEIK---------MDYKKNANVIVGTETIEEAIRQLMD-----PENEIRV
Si_UGT94D1 375 RLVEA-VGFGEEVV----------RSRQG---NLDRGEVARVVKKLVM---GKSGEGLRR
At_UGT71B6 389 FEMVEELGLAVEIK--KHWRGDLLLGRSE---IVTAEEIEKGIICLM-----EQDSDVRK
At_UGT73C6 403 KLVVQILKVGVSAEVKEVMKWGEEEKIGV---LVDKEGVKKAVEELMG--ESDDAKERRR
At_UGT73B1 403 KLVTQVLKTGVSVGVKKM-----MQVVGD---FISREKVEGAVREVMV------GEERRK
At_UGT75C1 381 KLVEDTWRIGVKVK----------VGEEG---DVDGEEIRRCLEKVMS--GGEEAEEMRE
At_UGT78D2 386 RAVEVVWEIGMTII-------------NG---VFTKDGFEKCLDKVLV---QDDGKKMKC
Gm_UGT73P2 397 KLVVDVLKIGVPVGAKEWRNW--NEFGSE---VVKREEIGNAIASLMS--EEEEDGGMRK
Gm_UGT91H4 394 RVLEE-KQVAVEVP---------RSEKDG---SFTRVDVAKTLRFAIV---DEEGSALRE
AcGaT 379 WMVENVWKIGVRIE-------------GG---VFTRTGTMNALEQVLL--SQEKGKKLKE
MtUGT85h2 408 RFICNEWEIGMEID-------------T----NVKREELAKLINEVIA---GDKGKKMKQ
GhUGT1 385 SYICNVWKIGLELQ------------------NLERGNIERTIKRLMV---DMEGKDIRK
AtUGT72B1 394 VLLSEDIRAALRPR----------AGDDG---LVRREEVARVVKGLME---GEEGKGVRN
VvUF3GT 380 RMVEDVLEIGVRIE-------------GG---VFTKSGLMSCFDQILS---QEKGKKLRE
MtUGT71g1 387 FRLVKEWGVGLGLR---------VDYRKGSD-VVAAEEIEKGLKDLMD-----KDSIVHK
MtUGT78g1 382 ILTESVLEIGVGVD-------------NG---VLTKESIKKALELTMS---SEKGGIMRQ
PL_ZOG1 382 VLVTEVLKVGLVVK-----DW---AQRNS---LVSASVVENGVRRLM---ETKEGDEMRQ
MtUGT72L1 400 VLVTEGLKVGLRPR----------VNENG---IVERVEVAKVIKRLME---GEECEKLHN
MtUGT73F3 398 KLITQFRGIGVEVGATEWCTSG-VAERKK---LVSRDSIEKAVRRLMD--GGDEAENIRL
MtUGT73K1 389 KLVTEVHRIGVEVGAAEWSMSPYDAKKT----VVRAERIEKAVKKLMD--SNGEGGEIRK
MtUGT88E2 391 LVLVDEMKVALKVN----------QSENR---FVSGTELGERVKELME---SDRGKDIKE
MtUGT88E1 388 LILVQEWKVALELN----------ESKDG---FVSENELGERVKELME---SEKGKEVRE
MtUGT73C8 403 SFVVQILKVGVKIGVKSPMKWG-EEEDGV---LVKKEDIERGIEKLMD--ETSECKERRK
AtUGT73C5 403 KLVVEVLKAGVRSGVEQPMKWGEEEKIGV---LVDKEGVKKAVEELMG--ESDDAKERRR
AtUGT73C1 399 KLAVQILKAGVRAGVEESMRWGEEEKIGV---LVDKEGVKKAVEELMG--DSNDAKERRK
AtUGT76C1 385 RFISEVWRVGIHLE--------------G---RIERREIERAVIRLMV---ESKGEEIRG
AtUGT76C2 378 RFVSDIWKIGIHLE--------------G---RIEKKEIEKAVRVLME---ESEGNKIRE
AtUGT85A1 411 KFCCDEWDVGIEIG--------------G---DVKREEVEAVVRELMD---GEKGKKMRE
Va_AOG 387 KLITEVLKTGVQVGNREWWPW--NAEWKG---LVGREKVEVAVRKLMV--ESVEADEMRR

GhUGT73C14 445 RAKELGDEANKAVEVG-GSSYMNITLLIQD-ILQQSQEMR-------------
Zm_iaglu 435 AAGEWRDRARAAVAPG-GSSDRNLDEFVQF-VRAGATEK--------------
Gtr_UGT78B1 419 NVGRLKEKAKDAVKAN-GSSTRNFESLLAA-FNKLDS----------------
Nt_UGT73A1 439 RAKAYKEMARKAIEEG-GSSYTGLTTLLED-ISTYSSTGH-------------
Pv_ZOX1 423 RAVKLKDEIHRSMDEG-GVSRMEMASFIAH-ISR-------------------
Pf_A5GT 428 NAIKWKTLAREAMGED-GSSLKNLNAFLHQ-VARA------------------
Sb_HMNGT 456 KAAEWKAAAEAAARKG-GASWRNVERVVNDLLLVGGKQ---------------
Ph_F3GalT 418 QIGMYKELALNAVGPS-GSSAENFKKLVDI-ITSCN-----------------
Db_B5GT 451 RAKKLKEMAWRAIEEG-GSSYSDLSALIEE-LKGYHTSEKE------------
Cm_FG2RT 421 KANEISESMKKIGDAQ-------MSVVVEK-LLQLVKKSE-------------
BpUGT94B1 406 KAKELGEIMKKNMEAE-------VDGIVIENLVKLCEMNN-------------
Ph_3RT 439 NQKKWKEFLLNKDIQS-----KYIGNLVNE-MTAMAKVSTT------------
Si__UGT71A9 442 KVRALKEKSRMALMEG-GSSYNYLKRFVENVVNNIS-----------------
Si_UGT94D1 418 RVEELSEKMREKGEEEIDSLVEELVTVVRR-RERSNLKSENSMKKLNVMDDGE
At_UGT71B6 439 RVNEISEKCHVALMDG-GSSETALKRFIQD-VTENIAWSETES----------
At_UGT73C6 458 RAKELGESAHKAVEEG-GSSHSNITFLLQD-IMQLAQSNN-------------
At_UGT73B1 449 RAKELAEMAKNAVKEG-GSSDLEVDRLMEE-LTLVKLQKEKV-----------
At_UGT75C1 426 NAEKWKAMAVDAAAEG-GPSDLNLKGFVDE-DE--------------------
At_UGT78D2 427 NAKKLKELAYEAVSSK-GRSSENFRGLLDA-VVNII-----------------
Gm_UGT73P2 450 RAKELSVAAKSAIKVG-GSSHNNMKELIRE-LKEIKLSKEAQETAPNP-----
Gm_UGT91H4 438 NAKEMGKVFSSEELHN-----KYIQDFIDA-LQKYRIPSAS------------
AcGaT 421 QITVFKELALKAVGPN-GSSTQNFKRLLEV-ITT-------------------
MtUGT85h2 448 KAMELKKKAEENTRPG-GCSYMNLNKVIKDVLLKQN-----------------
GhUGT1 424 RAMDLKKKAALCLMED-GST-SSFNGLIKQ-ITVSET----------------
AtUGT72B1 438 KMKELKEAACRVLKDD-GTSTKALSLVALK-WKAHKKELEQNGNH--------
VvUF3GT 421 NLRALRETADRAVGPK-GSSTENFITLVDL-VSKPKDV---------------
MtUGT71g1 432 KVQEMKEMSRNAVVDG-GSSLISVGKLIDD-ITGSN-----------------
MtUGT78g1 423 KIVKLKESAFKAVEQN-GTSAMDFTTLIQI-VTS-------------------
PL_ZOG1 428 RAVRLKNAIHRSMDEG-GVSHMEMGSFIAH-ISK-------------------
MtUGT72L1 444 NMKELKEVASNALKED-GSSTKTISQLTLK-WRNLVQKNQI------------
MtUGT73F3 452 RAREFGEKAIQAIQEG-GSSYNNLLALIDE-LKRSRDLKRLRDLKLDD-----
MtUGT73K1 443 RAKEMKEKAWKAVQEG-GSSQNCLTKLVDY-LHSVVVTKSVELN---------
MtUGT88E2 435 RILKMKISAKEARGGG-GSSLVDLKKLGDS-WREHASWNSLSPNSPFLLR---
MtUGT88E1 432 TILKMKISAKEARGGG-GSSLVDLKKLGDS-WREHASWTSVSPNSPFLFA---
MtUGT73C8 457 RIRELAEMAKKAVEKG-GSSHSNISLFIQD-IMKKNKDMMSSFIHGNANSK--
AtUGT73C5 458 RAKELGDSAHKAVEEG-GSSHSNISFLLQD-IMELAEPNN-------------
AtUGT73C1 454 RVKELGELAHKAVEEG-GSSHSNITFLLQD-IMQLEQPKK-------------
AtUGT76C1 425 RIKVLRDEVRRSVKQG-GSSYRSLDELVDR-ISIIIEPLVPT-----------
AtUGT76C2 418 RMKVLKDEVEKSVKQG-GSSFQSIETLANH-ILLL------------------
AtUGT85A1 451 KAVEWQRLAEKATEHKLGSSVMNFETVVSK-FLLGQKSQD-------------
Va_AOG 440 RAKDIAGKAARAVEEG-GTSYADVEALIQE-LQARTCANQG------------
